# Comparative efficacy of different exercise methods to improve cardiopulmonary function in stroke patients: a network meta-analysis of randomized controlled trials

**DOI:** 10.3389/fneur.2024.1288032

**Published:** 2024-01-17

**Authors:** Chengshuo Wang, Yanan Xu, Linli Zhang, Weijiao Fan, Zejian Liu, Mingjin Yong, Liang Wu

**Affiliations:** ^1^Tianjin Key Laboratory of Exercise Physiology and Sports Medicine, Institute of Sport, Exercise & Health, Tianjin University of Sport, Tianjin, China; ^2^Beijing Xiaotangshan Hospital, Beijing, China; ^3^Department of Rehabilitation, Lianyungang Hospital of Traditional Chinese Medicine, Lianyungang, China

**Keywords:** stroke, exercise, cardiopulmonary function, randomized controlled trials, network meta-analysis

## Abstract

**Background:**

Although some studies have shown that exercise has a good effect on improving the cardiopulmonary function of stroke patients, it still needs to be determined which exercise method does this more effectively. We, therefore, aimed to evaluate the effectiveness of different exercise methods in improving cardiovascular function in stroke patients through a network meta-analysis (NMA), providing a basis to select the best treatment plan for stroke patients.

**Methods:**

We systematically searched CNKI, WanFang, VIP, CBM, PubMed, Embase, Web of Science, and The Cochrane Library databases from establishment to 30 April 2023. Randomized controlled trials (RCT_S_) on exercise improving cardiopulmonary function in stroke patients were included, and we screened the included articles and extracted the relevant data. RevMan (version 5.4) and Stata (version 17.0) were used for data analysis.

**Results:**

We included 35 RCTs and a total of 2,008 subjects. Intervention measures included high-intensity interval training (HIIT), aerobic training (AT), resistance training (RT), combined aerobic and resistance exercise (CE), and conventional therapy (CT). In the network meta-analysis, the surface under the cumulative ranking area (SUCRA) ranking result indicated that HIIT improved peak oxygen uptake (VO_2peak_) and 6 mins walking distance (6MWD) optimally, with rankings of HIIT (100.0%) > CE (70.5%) > AT (50.2%) > RT (27.7%) > CT (1.6%), and HIIT (90.9%) > RT (60.6%) > AT (48.9%) > RT (48.1%) > CT (1.5%), respectively. The SUCRA ranking result showed that CE improved systolic blood pressure (SBP) and diastolic blood pressure (DBP) optimally, with rankings of CE (82.1%) > HIIT (49.8%) > AT (35.3%) > CT (32.8%), and CE (86.7%) > AT (45.0%) > HIIT (39.5%) > CT (28.8%), respectively.

**Conclusion:**

We showed that exercise can effectively improve the cardiopulmonary function of stroke patients. HIIT was the most effective in improving VO_2peak_ and 6MWD in stroke patients. CE was the most effective in improving SBP and DBP in stroke patients. However, due to the limitations of existing clinical studies and evidence, larger sample size, multi-center, and high-quality RCTs are needed to verify the above conclusions in the future.

**Systematic review registration:**

https://www.crd.york.ac.uk/prospero/, identifier [CRD42023436773].

## 1 Introduction

Stroke, also known as a cerebrovascular accident, is an acute cerebrovascular disease characterized by focal neurological deficits caused by various vascular causes (such as ischemia or hemorrhage) (1). The absolute number of incident strokes globally increased by 70.0% from 1990 to 2019, whereas prevalent strokes increased by 85.0% and deaths from stroke increased by 43.0% (2). Stroke has become the second leading cause of death globally after ischemic heart disease (3). The common functional disorders in stroke patients include motor, sensory, cardiopulmonary, speech, and swallowing (4).

Stroke patients typically exhibit varying degrees of impaired cardiopulmonary function. Peak aerobic capacity (VO_2peak_) is the highest level of oxygen consumption (VO_2_) attained during a graded exercise test (5). VO_2peak_ levels in stroke patients may drop 8–22 mL/kg/min, approximately 53% compared to the average age and sex-matched population (6). VO_2peak_ levels required for independent living in healthy people is 15–18 mL/kg/min (7), and very low VO_2peak_ levels after stroke may prohibit patients from performing higher levels of ADL and limit the sustainability of lower levels of ADL (8). In addition, maintaining cardiovascular health is essential to reduce the risk of recurrent stroke (9). Therefore, improving the cardiopulmonary function of stroke patients as soon as possible has important clinical significance for functional recovery and quality of life improvement.

After the stroke, exercise is an essential component in reducing the risk of future cardiovascular events and stroke recurrence (10), and there is increasing evidence that exercise has substantial benefits in improving cardiopulmonary function and musculoskeletal health in stroke patients. The Chinese Stroke Association guidelines for clinical management of cerebrovascular disorders recommend individualized exercise rehabilitation training for stroke survivors to improve cardiopulmonary function (Class I recommendation, Level B evidence) (11). The current exercise methods applied to stroke patients mainly include high-intensity interval training (HIIT), aerobic training (AT), resistance training (RT), and combined aerobic and resistance exercise (CE). HIIT is an efficient method of exercise that involves performing a high-intensity workout in a short period and actively recovering or resting during exercise (12). AT refers to the exercise carried out by the body with sufficient oxygen supply, mainly focused on aerobic metabolism (13). RT is an active movement of muscles relying on their strength to overcome external resistance (14), whereas CE refers to the combination of aerobic exercise and strength training. Conventional therapy (CT) refers to routine treatment and care, and the patient does not perform any regular exercise.

Scholars worldwide have explored different interventions, but the most effective and safe interventions to improve the cardiopulmonary function of stroke patients have yet to be concluded. Pairwise meta-analysis uses CT as the control, which cannot compare the treatment effects of multiple interventions. Network meta-analysis (NMA) was developed from the pairwise meta-analysis, from comparing two standard treatment factors to comparing numerous treatment factors simultaneously. The primary function of NMA is to evaluate and rank multiple interventions simultaneously (15). Therefore, we aimed to use NMA to assess and compare the effects of different exercise methods on improving cardiopulmonary function in stroke patients to provide sufficient evidence for future clinical practice.

## 2 Materials and methods

### 2.1 Study enrollment and reporting

This study was conducted following the recommendations of the Preferred Reporting Items for Systematic Reviews and Meta-Analyses (PRISMA) statement (16). PRISMA extension statements were used to ensure that all aspects of methods and results were reported (17). The protocol is registered in PROSPERO (registration number: CRD42023436773).

### 2.2 Search strategy

Two authors separately searched for randomized controlled trials (RCTs) regarding exercise improving cardiopulmonary function in stroke patients from the China National Knowledge Infrastructure (CNKI), WanFang Knowledge Service Platform (WanFang), Chinese Scientific Journals Database (VIP), Chinese Biomedical Literature Service System (CBM), PubMed, Embase, Web of Science, and The Cochrane Library databases. The retrieval period started from the establishment of the database to 30 April 2023. By combining medical subject headings with free words using Boolean logic operators, we integrated the following terms for a comprehensive search: “stroke,” “apoplexy,” “hemiplegia,” “cerebrovascular disease,” “cerebral infarction,” “cerebral hemorrhage,” “sport,” “exercise,” “train,” “physical activity,” “resistance exercise,” “aerobic exercise,” “high-intensity interval training,” “random,” “randomized controlled trial,” and “RCT.” In addition, we manually screened the list of references in the relevant meta-analysis and reviews to minimize the omission of literature that meets the inclusion criteria. Taking PubMed search as an example, the details of the search strategy are shown in Supplementary Table S1.

### 2.3 Selection and exclusion criteria

The inclusion criteria were formulated according to the principles of Population, Intervention, Comparison, Outcome, and Study design (PICOS) (18). Eligible studies had to meet the following criteria: (1) population: adult stroke patients with stable vital signs, no cognitive impairment and movement contraindication, and with the consent of the patient and his family members; (2) intervention: HIIT, AT, RT, and CE; (3) comparison: the control group only received CT or any of the above interventions; (4) outcome: in the included article, at least one of the following results must be reported: peak oxygen uptake (VO_2peak_), 6 min walking distance (6MWD), systolic blood pressure (SBP), and diastolic blood pressure (DBP); (5) study design: randomized controlled trial. The exclusion criteria were as follows: (1) studies that do not specify the type of exercise intervention; (2) studies with unclear descriptions of participant age; (3) conference articles, reviews, dissertations, and non-RCT_S_ (e.g., case reports, observational studies, cross-sectional studies, and studies without a control group); (4) studies with more patients withdrawing midway; (5) studies that could not be downloaded; and (6) studies with incomplete outcome data and contacting the authors three times without response.

### 2.4 Study selection

Two authors (CW and YX) independently screened the article using EndNote X9 software. If there was any disagreement during the process, the decision was made through consultation or jointly with the third author (LZ). During article screening, we first used the duplicate check function of the software to eliminate any of the same articles. The title, abstract, and body of the literature were then read sequentially, and those that did not meet the inclusion criteria were eliminated. In case of missing important information, we contacted the corresponding authors of the literature by email or other means to ensure the completeness of the data.

### 2.5 Data extraction and quality assessment

Two authors (WF and ZL) independently reviewed all the articles and extracted the data. The extracted data includes basic publication information (first author’s name and country of origin), participant characteristics (age and sample size), intervention characteristics (type, intensity, duration, and period), and outcome measures (VO_2peak_, 6MWD, SBP, and DBP) at baseline and last observation, to observe their change scores. When there were disagreements during data extraction, the third author (MY) was involved in the discussion and decision-making. Two authors (WF and ZL) used the Cochrane Risk of Bias Tool to evaluate the included article in the following aspects: (I) random sequence generation; (ii) allocation concealment; (iii) blinding of participants and personnel; (iv) blinding of outcome assessment; (v) incomplete outcome data; (vi) selective reporting; (vii) other bias (19). The risk assessment was divided into three levels: “low risk,” “high risk,” and “unclear.” The evaluation process was carried out by two authors independently, and if there were any disputes in the process, the third author (MY) was consulted and a decision made together.

### 2.6 Statistical analysis

Odds ratio for binary variables and mean difference (MD) for continuous variable were used as the effect indicators, and the 95% confidence interval (CI) was provided for each effect size. For continuous variable indicators, we calculated the difference before and after treatment and the standard deviation according to the method provided in 16.1.3.2 of Cochrane Handbook 5.0.2 for statistical analysis. We used RevMan (version 5.4) for pairwise meta-analysis. The *p*-value of the chi-square test and the *I*^2^ index from the heterogeneity test were used to express the level of statistical heterogeneity. Different effect models were selected according to the level of heterogeneity of the test data. When the level of heterogeneity was low (*p* ≥ 0.1, *I*^2^ ≤ 50%), we selected the fixed effect model for analysis. Otherwise, a random effect model (*p* < 0.1, *I*^2^ > 50%) was used (20).

We used Stata (version 17.0) for all statistical analysis and various charts, such as network meta-analysis diagrams of eligible comparisons, the surface under the cumulative ranking area (SUCRA), funnel plot of publication bias, and so on (21). When there are closed loops between interventions, we first need to assess global inconsistency. When *p* > 0.05, the inconsistent model was not significant, and the consistent model was selected (22). We used a node-splitting approach to assess local inconsistency (23). At the same time, it is also necessary to evaluate the loop inconsistency and calculate the inconsistency factors (IF) and 95% confidence interval (CI) for each closed loop. If the lower limit of 95% CI included or was close to 0, the consistency between the direct comparison results and the indirect comparison results was good; otherwise, the closed loop was considered to have obvious inconsistency. If no closed loop was formed between the interventions, the consistency model was used for analysis directly. Intervention outcomes were ranked using the SUCRA. The closer SUCRA was to 100%, the better the effect of the intervention. Finally, the publication bias of the included articles was evaluated by drawing the funnel plot of publication bias and Egger’s test. Publication bias was indicated when there was asymmetry in the funnel plot of publication bias and *p* < 0.05 in Egger’s test (24).

## 3 Results

### 3.1 Study identification and selection

**Figure 1:**
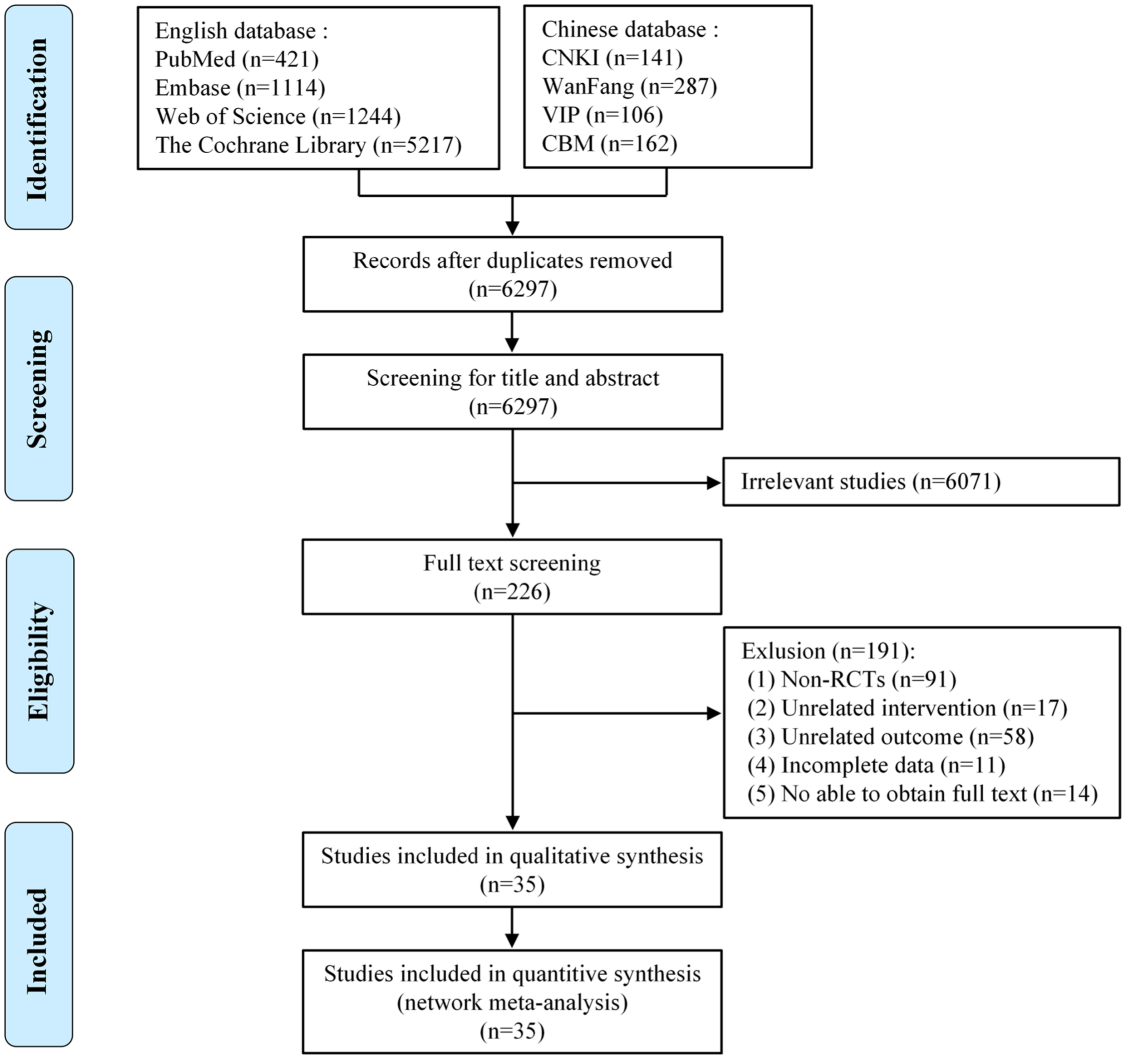
Flow diagram of eligible studies selection process. CNKI, China national knowledge infrastructure; WanFang, WanFang knowledge service platform; VIP, Chinese scientific journals database; CBM, Chinese biomedical literature service system; *n*, number of publications.

We strictly searched the above eight databases according to the inclusion and exclusion criteria and preliminarily obtained 8,692 articles. After eliminating duplicates, 6,297 articles remained. By reading the titles and abstracts of the articles, those that did not meet the inclusion criteria were excluded, leaving 226 articles. By reading the full text, we excluded a further 191 articles, including non-RCTs (*n* = 91), articles with unrelated intervention (*n* = 17), articles with irrelevant outcomes (*n* = 58), articles with unavailable full text (*n* = 14), and articles with incomplete data (*n* = 11). Ultimately, 35 articles met our study requirements (Figure 1).

### 3.2 Characteristics of the included studies

We finally included 35 RCTs with 1,075 patients in the intervention group and 933 patients in the control group, ranging in age from 55 to 78 years. The RCTs were from China (*n* = 9), the United States (*n* = 7), Canada (*n* = 4), South Korea (*n* = 4), Norway (*n* = 2), Ireland (*n* = 1), Denmark (*n* = 1), Switzerland (*n* = 1), Sweden (*n* = 1), Australia (*n* = 1), Italy (*n* = 1), Belgium (*n* = 1), Germany (*n* = 1), and Israel (*n* = 1). Among the 35 articles, one was a four-arm trial, three were three-arm trials, and 31 articles were two-arm trials. Twenty-nine articles used VO_2peak_ as the outcome measure; 21 used 6MWD as the outcome measure; and seven used SBP and DBP as the outcome measure. Table 1 shows the key characteristics of the patients and interventions included in this study.

**Table 1:** Characteristics of included studies.

Study ID	Country	Age (years)	Sample	Type of intervention	Intensity of intervention	Duration of intervention	Intervention period	Outcomes
Munari et al. (25)	Italy	HIIT: 61.0 ± 5.8	8	HIIT	HIIT: 85–95% HRR (5 min); Intervals active: 50% HRR (3 min); 5 groups	50–60 min	3 times per week for 12 weeks	VO_2peak_, 6MWD
		AT: 62.0 ± 11.3	7	AT	40–60% VO_2peak_	55 min		
Gjellesvik et al. (26)	Norway	HIIT: 57.6 ± 9.2	33	HIIT	HIIT: 85–95% HR_peak_; Intervals recovery (4 min); 4 groups	38 min	3 times per week for 8 weeks	VO_2peak_, 6MWD, SBP, DBP
		CT: 58.7 ± 9.2	31	CT	–	–	3–5 times per week for 8 weeks	
Gjellesvik et al. (27)	Norway	HIIT: 57.6 ± 9.2	33	HIIT	HIIT: 85–95% HR_peak_; Intervals recovery (4 min); 4 groups	38 min	3 times per week for 8 weeks	6MWD
		CT: 58.7 ± 9.2	31	CT	–	–	3–5 times per week for 8 weeks	
Boyne et al. (28)	America	HIIT: 59.0 ± 9.0	13	HIIT	HIIT: Started at 0.1 mph below maximum safe speed and held for 30 s; Intervals recovery (30–60s)	25 min	3 times per week for 4 weeks	VO_2peak_, 6MWD
		AT: 57.0 ± 12.0	5	AT	48–54% HRR	25 min		
Ivey et al. (29)	America	HIIT: 61.0 ± 1.6	24	HIIT	HIIT: 80–85% HRR; Intervals recovery	30 min	24 weeks	VO_2peak_, 6MWD
		AT: 63.0 ± 2.4	27	AT	<50% HRR	30 min		
Soh et al. (30)	Korea	HIIT: 56.3 ± 5.3	22	HIIT	HIIT: Borg <14 (1 min); Intervals recovery (60s)	30 min	3 times per week for 12 weeks	VO_2peak_, 6MWD, SBP, DBP
		AT: 57.4 ± 7.2	23	AT	50–80% HRR/Borg <14	30 min		
Hsu et al. (31)	China	HIIT: 58.5 ± 23.5	13	HIIT	HIIT: 80% VO_2peak_ (3 min); Intervals active: 40% VO_2peak_ (3 min); 5 groups	30 min	2–3 times per week for 12 weeks	VO_2peak_
		AT: 53.1 ± 18.6	5	AT	60% VO_2peak_	30 min		
Ye et al. (32)	China	HIIT: 58.9 ± 5.3	60	HIIT	HIIT: 80% W_max_ (3 min); Intervals recovery (60s); 10 groups	40 min	5 times per week for 12 weeks	VO_2peak_
		AT: 59.00 ± 4.64	60	AT	40% W_max_	40 min		
		CT: 60.20 ± 4.96	60	CT	–	–		
Sandberg et al. (33)	Sweden	HIIT: 71.3 ± 7.0	29	HIIT	HIIT: ≥75% VO_2peak_; Intervals active: ≥50% VO_2peak_	60 min	2 times per week for 12 weeks	6MWD
		CT: 70.4 ± 8.1	27	CT	–	60 min		
Globas et al. (34)	Germany	AT: 68.6 ± 6.7	20	AT	40–80% HRR	30–50 min	3 times per week for 12 weeks	VO_2peak_, 6MWD
		CT: 68.7 ± 6.1	18	CT	–	–		
Jin et al. (35)	China	AT: 57.6 ± 6.6	65	AT	40–70% HRR	40 min	5 times per week for 12 weeks	VO_2peak_, 6MWD, SBP, DBP
		CT: 56.3 ± 6.5	63	CT	–	40 min		
Xu et al. (36)	China	AT: 59 ± 12	15	AT	60% VO_2max_	30 min	5 times per week for 4 weeks	VO_2peak_
		CT: 64 ± 9	15	CT	–	30 min		
Han et al. (37)	China	AT: -	69	AT	60–80% HR_max_	30 min	5 times per week for 8 weeks	VO_2peak_, 6MWD
		CT: -	69	CT	–	30 min		
Bang et al. (38)	Korea	AT 56.8 ± 6.5	6	AT	50–80% HR_max_/Borg: 11–14	30 min	5 times per week for 4 weeks	6MWD
		CT: 63.7 ± 5.8	6	CT	–	30 min		
Rimmer et al. (39)	China	AT: 55.7 ± 12.6	18	AT	40–69% HRR	30 min	3 times per week for 14 weeks	VO_2peak_, SBP, DBP
		CT: 63.7 ± 9.1	18	CT	–	30 min		
Gu et al. (40)	China	AT: 66.5 ± 7.8	43	AT	40–60% VO_2peak_	30 min	5 times per week for 12 weeks	VO_2peak_, 6MWD
		CT: 64.1 ± 9.2	43	CT	–	30 min		
Tang et al. (41)	Canada	AT: 64.7 ± 3.6	23	AT	50–75% VO_2peak_	30 min	3 times per week for 4–5 weeks	VO_2peak_, 6MWD
		CT: 65.7 ± 2.3	22	CT	–	–	5 times per week for 4–5 weeks	
Macko et al. (42)	America	AT: 63 ± 10	32	AT	40–70% HRR	30–40 min	3 times per week for 24 weeks	VO_2peak_, 6MWD
		CT: 64 ± 8	29	CT	–	35 min		
Mackay-Lyons et al. (43)	Canada	AT: 61.5 ± 15.4	24	AT	40–75% VO_2peak_	60 min	5 times per week for 12 weeks	VO_2peak_, 6MWD
		CT: 59.0 ± 12.7	26	CT	–	60 min		
Vanroy et al. (44)	Belgium	AT: 66.7 ± 8.8	33	AT	60–80% HR_max_	30 min	3 times per week for 12 weeks	VO_2peak_
		CT: 63.8 ± 11.8	26	CT	–	30 min		
Tang et al. (45)	Canada	AT: 65.9 ± 6.4	25	AT	40–80% HRR/Borg: 11–14	60 min	3 times per week for 24 weeks	VO_2peak_, 6MWD
		CT: 66.9 ± 7.8	25	CT	<40% HRR	60 min		
Severinsen et al. (46)	Denmark	AT: 69 ± 24.82	17	AT	50–75% HRR	60 min	3 times per week for 12 weeks	VO_2peak_, 6MWD
		RT: 68 ± 17.38	14	RT	50–80% 1RM	–		
		CT: 66 ± 23.17	17	CT	–	–		
Chang et al. (47)	Korea	AT: 55.5 ± 12.0	24	AT	speed starting at 1.2 km/h, gradually increased to 2.6 km/h	40 min	5 times per week for 2 weeks	VO_2peak_
		CT: 59.7 ± 12.1	24	CT	–	–		
Lennon et al. (48)	Ireland	AT: 60.5 ± 10.0	24	AT	50–60% HR_max_	30 min	10 weeks	VO_2peak_, SBP, DBP
		CT: 59.0 ± 10.3	24	CT	–	–		
Stoller et al. (49)	Switzerland	AT: 57.0 ± 12.0	7	AT	40–70% HRR	30 min	3 times per week for 4 weeks	VO_2peak_
		CT: 63.0 ± 13.0	7	CT	–	–		
Fu et al. (50)	China	AT: 77.5 ± 6.5	20	AT	Borg: 10–12	30 min	5 times per week for 8 weeks	6MWD
		CT: 78.8 ± 6.8	20	CT	–	30 min		
Ivey et al. (51)	America	RT: 57 ± 14	22	RT	70% 1RM	45 min	3 times per week for 12 weeks	VO_2peak_, 6MWD
		CT: 55 ± 9	16	CT	–	45 min		
Shao et al. (52)	China	RT: 64.56 ± 7.08	69	RT	–	45 min	5 times per week for 6 weeks	6MWD
		CT: 65.72 ± 5.95	70	CT	–	45 min		
Marzolini et al. (53)	Canada	CE: 61.7 ± 10.0	36	CE	AT: 60–80% HRR/VO_2peak_; RT: 50–70% 1RM	AT: 20–60 min; RT: -	AT: 3 times per week for 6 weeks; RT: 2 times per week for 6 weeks	VO_2peak_, 6MWD, SBP, DBP
		AT: 65.6 ± 13.2	38	AT	60–80% HRR/VO_2peak_	20–60 min	5 times per week for 6 weeks	
Ran et al. (54)	China	CE: -	20	CE	AT: Borg: 12–15; RT: Borg: 12–15	AT: 20 min; RT: 20 min	5 times per week for 4 weeks	VO_2peak_
		AT: -	20	AT	Borg: 12–15	40 min		
Carr et al. (55)	America	CE: -	20	CE	AT: 40–70% W_max_; RT: -	AT: 20–40 min RT: -	3 times per week for 16 weeks	VO_2peak_
		AT: -	20	AT	40–70% W_max_	20–40 min		
Lee et al. (56)	Canada	CE: 60.5 ± 10.6	13	CE	AT: 50–70% VO_2peak_; RT: 50–80% 1RM	30 min	3 times per week for 10–12 weeks	VO_2peak_, 6MWD
		RT: 62.9 ± 9.3	13	RT	50–80% 1RM	30 min		
		AT: 67.2 ± 10.6	14	AT	50–70% VO_2peak_	30 min		
		CT: 65.3 ± 6	12	CT	-	30 min		
Kang et al. (57)	Korea	CE: 52.55 ± 3.28	15	CE	AT: 50–70% HR_max_; RT: Borg: 12–13	60 min	3 times per week for 8 weeks	VO_2peak_
		RT: 56.43 ± 2.08	15	RT	Borg: 12–13	60 min		
		CT: 56.36 ± 2.76	15	CT	–	60 min		
Duncan et al. (58)	America	CE: 68.5 ± 9.0	50	CE	AT: 40 rpm; RT: -	AT: 30 min; RT: -	3 times per week for 8 weeks	VO_2peak_, 6MWD
		CT: 70.2 ± 11.4	50	CT	–	–		
Toledano-Zarhi A et al. (59)	Switzerland	CE: 65 ± 10	14	CE	AT: 50–70% HR_max_; RT: -	AT: 35–55 min; RT: 45–55 min	2 times per week for 8 weeks	6MWD, SBP, DBP
		CT: 65 ± 12	14	CT	–	–		

HIIT, high-intensity interval training; AT, aerobic training; RT, resistance training; CE, combined aerobic and resistance exercise; CT, conventional therapy; HRR, heart rate reserve; VO_2peak_, peak oxygen uptake, HR_max_, maximum heart rate; Borg, Borg scale; 1RM, one repetition maximum; 6MWD, 6 min walking distance; SBP, systolic blood pressure; DBP, diastolic blood pressure; rpm, revolutions per minute; HR_peak_, peak heart rate; −, not reported.

### 3.3 Quality evaluation

**Figure 2:**
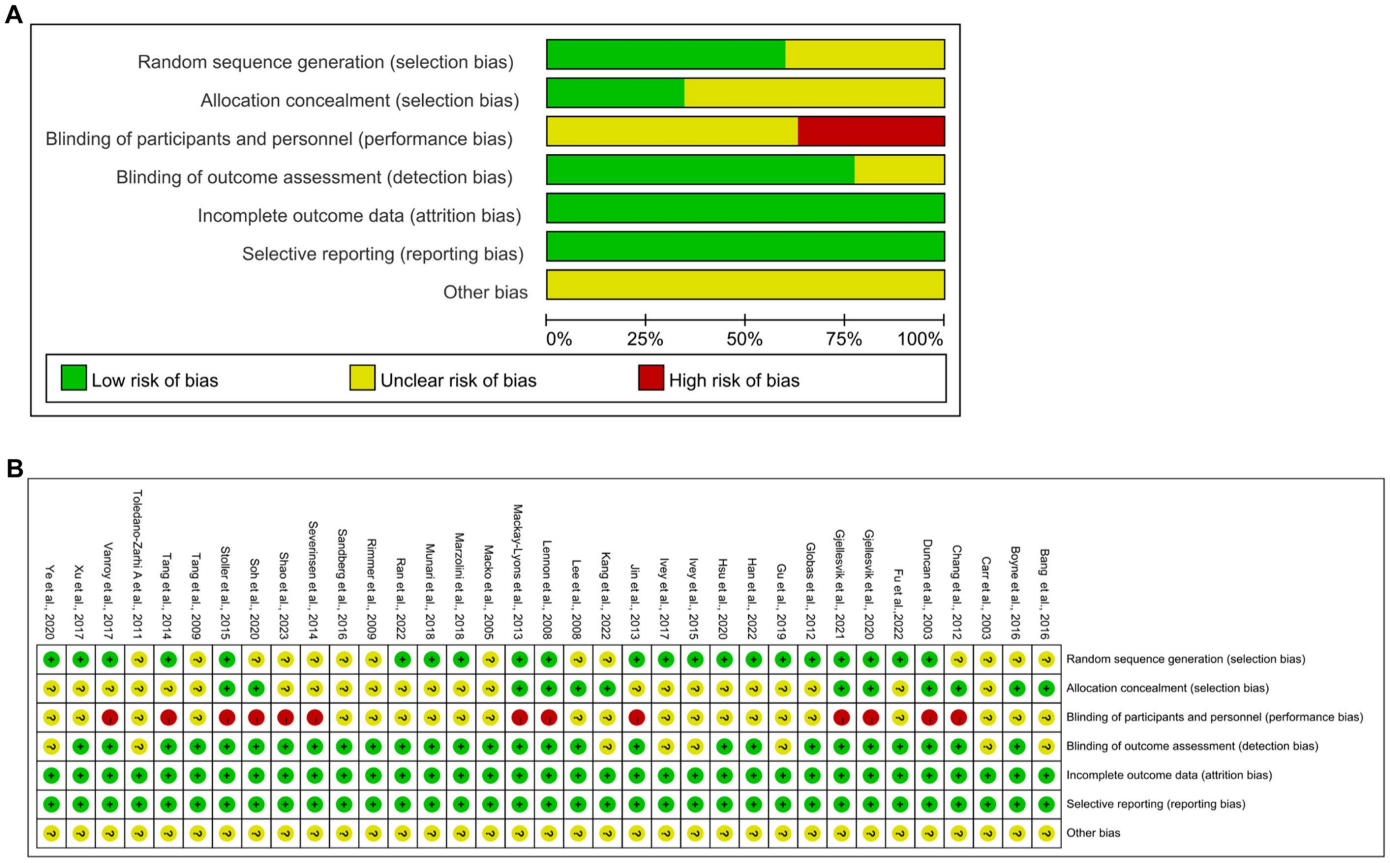
Quality assessment of selected studies by the cochrane risk of bias tool. **(A)** Risk of bias graph: review authors judgments about each risk of bias item presents as percentages across all included studies. **(B)** Risk of bias summary: review authors judgements about each risk of bias item for each included study.

All 35 articles included were RCTs. Twenty-one articles reported random sequence generation, rated as a low risk of bias; 14 did not adequately report how randomization was performed and were rated as uncertain risk of bias; 12 described allocation concealment and were rated as having a low risk of bias; 22 did not fully report blinding of researchers and subjects, rated as an uncertain risk of bias; 13 did not blind the investigators and subjects and were rated as a high risk of bias; and eight articles described the blinding of outcome measures and were rated as having a low risk of bias. None of the remaining articles were reported and rated as having an uncertain risk of bias. All 35 articles showed good data integrity and did not report the study results selectively. Furthermore, all articles did not describe any other bias. Figure 2 shows the details of the bias risk assessment results.

### 3.4 Pairwise meta-analysis

In this study, we used a pairwise meta-analysis to comprehensively compare two interventions. We carried out eight pairwise meta-analyses to compare VO_2peak_, 8 to compare 6MWD, 5 to compare SBP, and 5 to compare DBP, respectively, which can be summarily seen in Table 2. The detailed forest plots of the pairwise meta-analysis results were shown in Supplementary Figures S1–S4.

**Table 2:** Pairwise meta-analysis.

Comparison	Number of studies	MD (95% CI)	Heterogeneity test	
			*I*^2^ (%)	*p*-value
VO_2peak_				
HIIT – CT	2	**3.61 (3.08, 4.15)**	0	0.66
AT – CT	17	**1.97 (1.29, 2.65)**	78	<0.00001
RT – CT	4	**1.68 (0.09, 3.26)**	66	0.03
CE – CT	3	3.15 (−0.12, 6.42)	80	0.006
HIIT – AT	6	**3.57 (2.23, 4.91)**	74	0.002
AT – RT	2	**1.92 (0.42, 3.43)**	0	0.96
RT – CE	2	−2.16 (−4.99, 0.66)	0	0.52
AT – CE	4	−1.12 (−2.41, 0.16)	0	0.51
6MWD				
HIIT – CT	2	**61.74 (19.63, 103.86)**	0	0.64
AT – CT	10	**32.90 (2.89, 62.91)**	74	<0.0001
RT – CT	5	**38.19 (3.28, 73.10)**	79	0.0007
CE – CT	3	**27.66 (3.31, 52.01)**	0	0.99
HIIT – AT	3	30.08 (−6.79, 66.96)	49	0.14
AT – RT	2	−9.26 (−45.32, 26.79)	0	0.81
AT – CE	2	−3.88 (−31.45, 23.70)	0	0.89
RT – CE	1	−16.80 (−127.13, 93.53)	–	–
SBP				
HIIT – CT	1	0.08 (−7.08, 7.24)	–	–
AT – CT	3	−0.57 (−4.91, 3.76)	0	0.52
CE – CT	1	−3.20 (−15.30, 8.90)	–	–
HIIT – AT	1	−1.30 (−6.59, 3.99)	–	–
AT – CE	1	3.50 (−3.93, 10.93)	–	–
DBP				
HIIT – CT	1	0.28 (−4.44, 5.00)	–	–
AT – CT	3	−1.51 (−4.40, 1.37)	46	0.16
CE – CT	1	−0.10 (−5.48, 5.28)	–	–
HIIT – AT	1	−0.40 (−5.43, 4.63)	–	–
AT – CE	1	3.70 (−0.66, 8.06)	–	–

HIIT, high-intensity interval training; AT, aerobic training; RT, resistance training; CE, combined aerobic and resistance exercise; CT, conventional therapy; −, not reported. Red and bold numbers are statistically significant.

### 3.5 Network analysis results

#### 3.5.1 VO_2peak_

**Figure 3:**
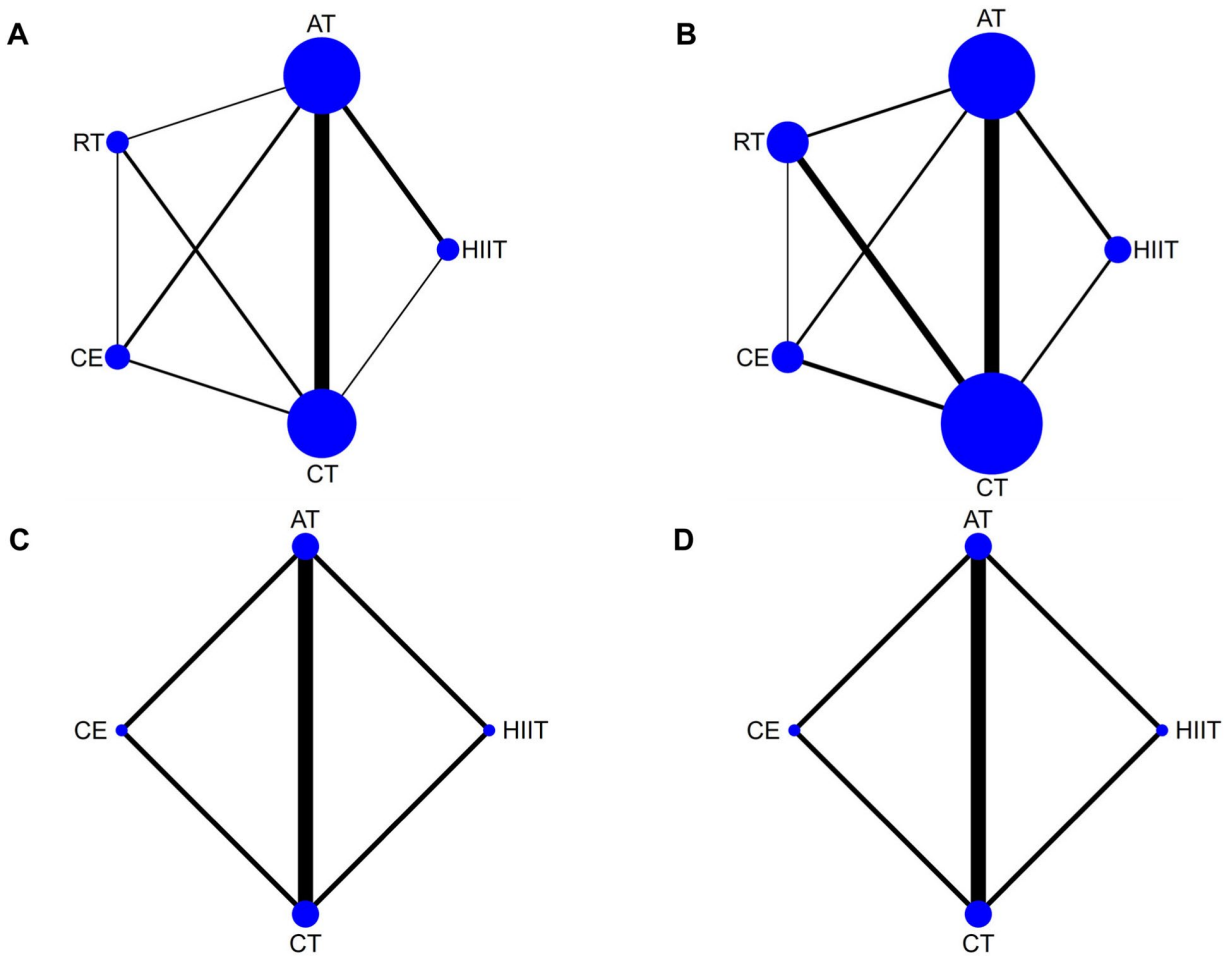
Network meta-analysis diagrams of eligible comparisons. Width of the lines is proportional to the number of trial. Size of every circle is proportional to the number of randomly assigned participants (sample size). **(A)** VO_2peak_; **(B)** 6WMD; **(C)** SBP; **(D)** DBP. HIIT, high-intensity interval training; AT, aerobic training; RT, resistance training; CE, combined aerobic and resistance exercise; CT, conventional therapy.

VO_2peak_ was reported in 29 articles involving five interventions: HIIT, AT, RT, CE, and CT with a total of 1,534 patients. Figure 3A shows the NMA diagrams of eligible comparisons, and the blue dots represent different interventions. The size of the dots represents the sample size; the straight line between two dots represents a direct comparison between two various interventions; and the thicker the solid line indicates the more significant number of studies in that pairwise comparison.

The inconsistency model evaluated global inconsistency, which showed *p* = 0.069 (>0.05) (Supplementary Figure S5A). The inconsistency test was not significant, so we used the consistency model. We used the node-splitting approach to assess local inconsistency and only measured *p* < 0.05 for HIIT compared with CT (Supplementary Table S2). Nine closed loops were formed for the five interventions, and we assessed loop inconsistency for all closed loops. The results showed that all the 95% CI included 0, and all the IF were close to 0, indicating that the statistical results of NMA were highly credible (Supplementary Figure S6A).

**Figure 4:**
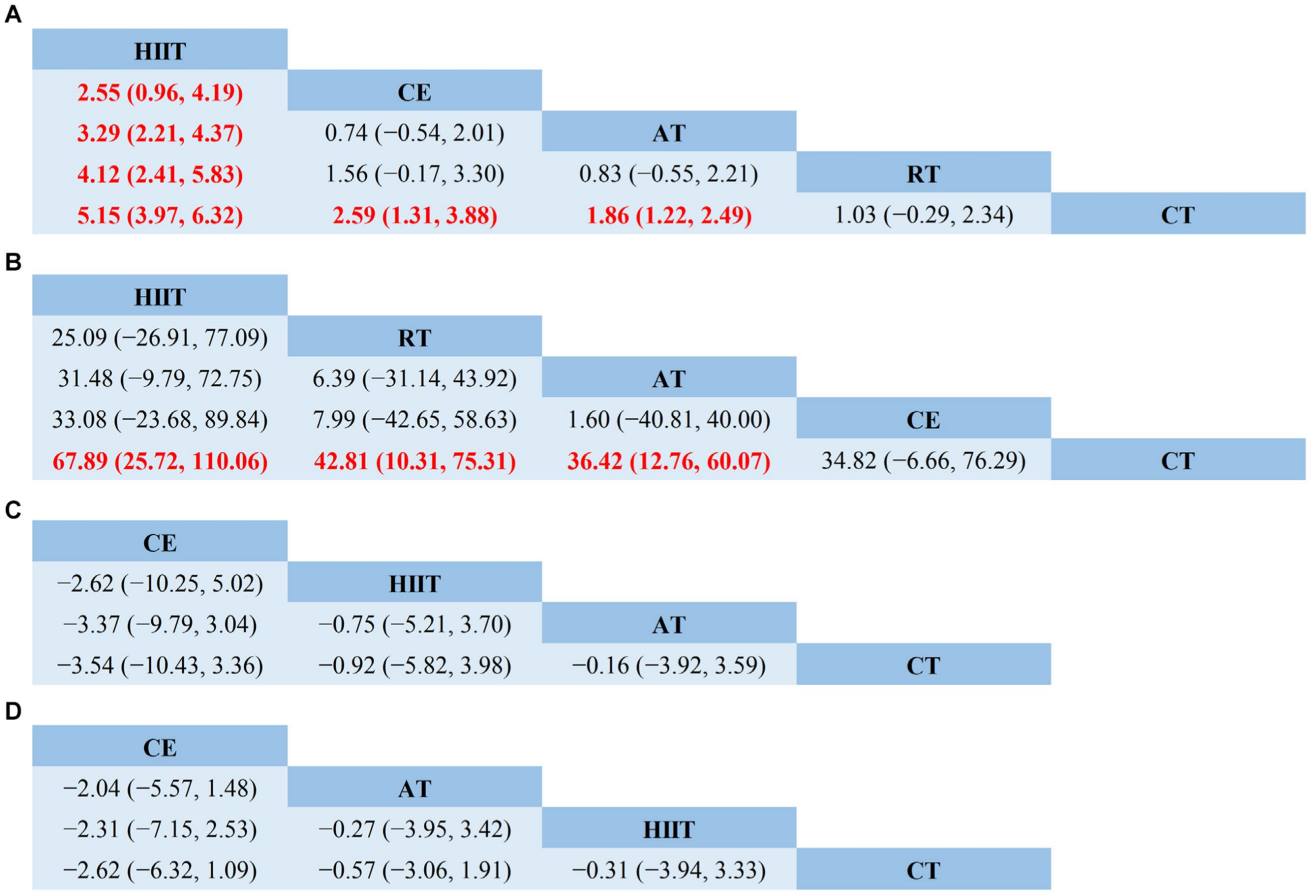
Network meta-analysis of head-to-head comparisons. **(A)** VO_2peak_; **(B)** 6WMD; **(C)** SBP; **(D)** DBP. Red and bold numbers are statistically significant. HIIT, high-intensity interval training; AT, aerobic training; RT, resistance training; CE, combined aerobic and resistance exercise; CT, conventional therapy.

The NMA results showed that VO_2peak_ generated a total of 10 pairwise comparisons. HIIT significantly improved VO_2peak_ compared to CE (MD = 2.55, 95% CI [0.96, 4.19]), AT (MD = 3.29, 95% CI [2.21, 4.37]), and RT (MD = 4.12, 95% CI [2.41, 5.83]). Compared with CT, HIIT (MD = 5.15, 95% CI [3.97, 6.32]), CE (MD = 2.59, 95% CI [1.31, 3.88]), and AT (MD = 1.86, 95% CI [1.22, 2.49]) significantly improved VO_2peak_ in stroke patients. There was no statistically significant difference between the other two interventions (*p* > 0.05) (Figure 4A). Table 3 showed the SUCRA ranking for all interventions. According to the analysis, HIIT (SUCRA, 100.0%) may be the most effective intervention to improve VO_2peak_ in stroke patients, followed by CE (SUCRA, 70.5%), AT (SUCRA, 50.2%), RT (SUCRA, 27.7%), and CT (SUCRA, 1.6%).

**Table 3:** SUCRA ranking of different interventions of outcomes.

Type of intervention	VO_2peak_			6MWD			SBP			DBP		
	SUCRA, %	Rank, %	Mean rank	SUCRA, %	Rank, %	Mean rank	SUCRA, %	Rank, %	Mean rank	SUCRA, %	Rank, %	Mean rank
HIIT	100.0	99.9	1.0	90.9	75.1	1.4	49.8	19.4	2.5	39.5	14.4	2.8
AT	50.2	0.0	3.0	48.9	1.9	3.0	35.3	4.3	2.9	45.0	7.4	2.6
RT	27.7	0.0	3.9	60.6	14.2	2.6	–	–	–	–	–	–
CE	70.5	0.1	2.2	48.1	8.8	3.1	82.1	70.2	1.5	86.7	74.6	1.4
CT	1.6	0.0	4.9	1.5	0.0	4.9	32.8	6.1	3.0	28.8	3.6	3.1

Higher SUCRA and lower mean rank indicate a better intervention effect. HIIT, high-intensity interval training; AT, aerobic training; RT, resistance training; CE, combined aerobic and resistance exercise; CT, conventional therapy; −, not reported.

#### 3.5.2 6MWD

6MWD was reported in 21 articles involving five interventions: HIIT, AT, RT, CE, and CT, with a total of 1,156 patients. Figure 3B shows the NMA diagrams of eligible comparisons. The inconsistency model evaluated global inconsistency, which showed *p* = 0.6592 (>0.05) (Supplementary Figure S5B). The inconsistency test was not significant, so the consistency model was used.

The node-splitting approach was used to evaluate local inconsistency. The measured *p*-values were all >0.05, indicating good local consistency (Supplementary Table S3). Nine closed loops were formed for the five interventions, and we assessed loop inconsistency for all closed loops. The results showed that all the 95% CI included 0, and all the IF were close to 0, indicating that the statistical results of NMA were highly credible (Supplementary Figure S6B). The NMA results showed that 6MWD generated a total of 10 pairwise comparisons. Compared with CT, HIIT (MD = 67.89, 95% CI [25.72, 110.06]), RT (MD = 42.81, 95% CI [110.31, 75.31]), and AT (MD = 36.42, 95% CI [12.76, 60.07]) can significantly improve 6MWD in stroke patients. There was no statistically significant difference between the other two interventions (*p* > 0.05) (Figure 4B). Table 3 showed the SUCRA ranking for all interventions. According to the results of the SUCRA analysis, HIIT (SUCRA, 90.9%) may be the most effective intervention to improve 6MWD in stroke patients, followed by RT (SUCRA, 60.6%), AT (SUCRA, 48.9%), CE (SUCRA, 48.1%), and CT (SUCRA, 1.5%).

#### 3.5.3 Systolic blood pressure

SBP was reported in seven articles involving four interventions: HIIT, AT, CE, and CT with a total of 394 patients. Figure 3C shows the NMA diagrams of eligible comparisons. The inconsistency model evaluated global inconsistency, which showed *p* = 0.9259 (>0.05) (Supplementary Figure S5C). The inconsistency test was not significant, so the consistency model was used.

The node-splitting approach was used to evaluate local inconsistency. The measured *p*-values were all >0.05, indicating good local consistency (Supplementary Table S4). Three closed loops were formed for the four interventions, and we assessed loop inconsistency for all closed loops. The results showed that all the 95% CI included 0, and all the IF were close to 0, indicating that the statistical results of NMA were highly credible (Supplementary Figure S6C). The NMA results showed that SBP generated a total of six pairwise comparisons and there was no statistically significant difference between the pairwise comparisons (*p* > 0.05) (Figure 4C). Table 3 showed the SUCRA ranking for all interventions. According to the results of the SUCRA analysis, CE (SUCRA, 82.1%) may be the most effective intervention to improve SBP in stroke patients, followed by HIIT (SUCRA, 49.8%), AT (SUCRA, 35.3%), and CT (SUCRA, 32.8%).

#### 3.5.4 Diastolic blood pressure

DBP was reported in seven articles involving four interventions: HIIT, AT, CE, and CT with a total of 394 patients. Figure 3D shows the NMA diagrams of eligible comparisons. The inconsistency model evaluated global inconsistency, which showed *p* = 0.5571 (>0.05) (Supplementary Figure S5D). The inconsistency test was not significant, so the consistency model was used.

The node-splitting approach was used to evaluate local inconsistency. The measured *p*-values were all greater than 0.05, indicating good local consistency (Supplementary Table S5). Three closed loops were formed for the four interventions, and we assessed loop inconsistency for all closed loops. The results showed that all the 95% CI included 0, and all the IF were close to 0, indicating that the statistical results of NMA were highly credible (Supplementary Figure S6D). The NMA results showed that SBP generated a total of six pairwise comparisons, and there was no statistically significant difference between the pairwise comparisons (*p* > 0.05) (Figure 4D). Table 3 showed the SUCRA ranking for all interventions. According to the results of the SUCRA analysis, CE (SUCRA, 86.7%) may be the most effective intervention to improve DBP in stroke patients, followed by AT (SUCRA, 45.0%), HIIT (SUCRA, 39.5%), and CT (SUCRA, 28.8%).

### 3.6 Publication bias

**Figure 5:**
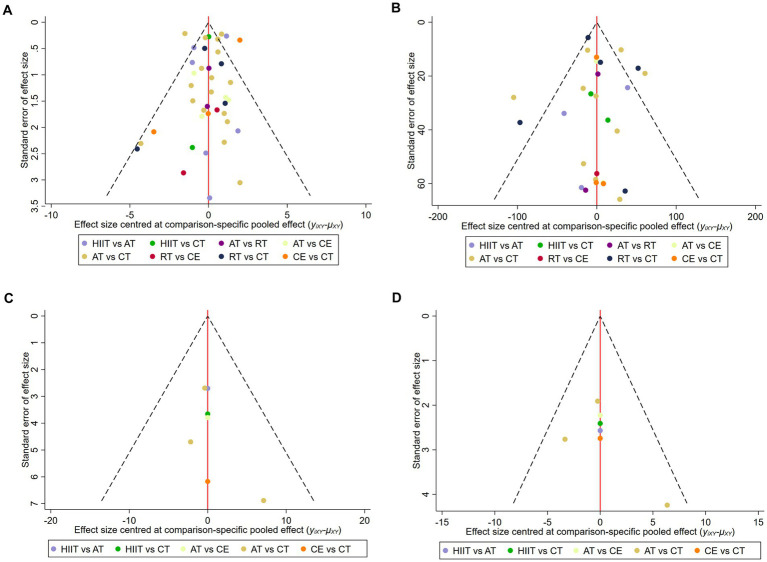
Funnel plot of publication bias. **(A)** VO_2peak_; **(B)** 6WMD; **(C)** SBP; **(D)** DBP. HIIT, high-intensity interval training; AT, aerobic training; RT, resistance training; CE, combined aerobic and resistance exercise; CT, conventional therapy.

We evaluated publication bias for VO_2peak_, 6MWD, SBP, and DBP using the funnel plot of publication bias (Figure 5) and Egger’s test. The colored dots in the funnel plot of publication bias represent pairwise comparisons between two different interventions. The greater the number of dots, the greater the number of pairwise comparisons. The dots in our funnel plot of publication bias were generally symmetrically distributed and concentrated at the top of the funnel. However, a few dots were allocated on the outside of the funnel in Figure 5A, and a few dots were distributed below and outside the funnel in Figure 5B, indicating a possible publication bias. In addition, we used Egger’s test for secondary validation of publication bias. The results showed VO_2peak_ (Egger’s test *p* = 0.819) (Supplementary Figure S7A), 6MWD (Egger’s test *p* = 0.384) (Supplementary Figure S7B), SBP (Egger’s test *p* = 0.268) (Supplementary Figure S7C), and DBP (Egger’s test *p* = 0.812) (Supplementary Figure S7D), indicating that there was no publication bias in this study.

### 3.7 Safety assessment of exercise training

In most of the included studies, investigators strictly implemented safety measures to ensure patient safety. Fifteen studies reported no adverse events during the exercise intervention period. Nine studies reported adverse events during the intervention period (Supplementary Table S6), involving 60 patients and four interventions (HIIT, AT, RT, and CT). The main adverse events included falls, fractures after falls, pneumonia, seizures, recurrent stroke, lower limb deep vein thrombosis, joint or muscle pain, dizziness, unstable blood pressure, brain tumors, concussions, aortic aneurysms, hernias, traumatic bleeding, and death. No adverse events were reported in the remaining 11 studies. Because there was no significant difference in the incidence of adverse events between the intervention group and the control group (*p* > 0.05), we believe exercise intervention is feasible for stroke patients. Still, it should be noted that exercise intervention must be carried out under the guidance and supervision of professionals.

## 4 Discussion

After stroke, the decline of nervous system function, prolonged bed rest, and decreased exercise will seriously affect the cardiopulmonary circulation system of patients. Girard et al. (60) found that stroke patients were inactive for 21 to 80% of their time during inpatient rehabilitation. In addition, it is difficult for patients to achieve 40% HRR in routine rehabilitation treatment. Conventional rehabilitation therapy is not sufficient to produce the effect of cardiopulmonary training, which hinders the recovery of functional independence of patients and may increase the risk of future stroke and other cardiovascular events. Therefore, for stroke patients, additional exercise to improve cardiopulmonary function is critical and necessary throughout the rehabilitation process. Our NMA evaluated the relative effectiveness of different exercise methods in improving VO_2peak_, 6MWD, SBP, and DBP in stroke patients by analyzing data from RCTs. This NMA was based on 35 studies, including a total of 2,008 patients. Twenty-nine RCTs assessed the effectiveness of four exercise methods and CT in improving VO_2peak_ in stroke patients; 21 RCTs assessed the effects of four exercise methods and CT on 6MWD; seven RCTs assessed the effects of three exercise methods and CT on SBP and DBP. To our knowledge, this is the first NMA to compare the effects of different exercise methods on improving cardiopulmonary function in stroke patients. The NMA showed that HIIT was the most effective in improving VO_2peak_, followed by CE. HIIT was the most effective in improving 6MWD, followed by RT. CE was the most effective in improving SBP and DBP in stroke patients. HIIT and AT also improved blood pressure in stroke patients to varying degrees.

In published studies, VO_2peak_ is one of the most commonly used measures to assess cardiopulmonary function in stroke patients, is the gold standard to evaluate an individual’s cardiopulmonary fitness, and is negatively correlated with cardiovascular risk and all-cause mortality (61). 6MWD, which refers to the maximum distance a participant can walk in 6 min, is also a commonly used indicator to assess an individual’s aerobic capacity and exercise endurance and is reliable and valid for evaluating cardiopulmonary function in stroke patients (62). This study shows that HIIT is the most potential exercise method to improve VO_2peak_ and 6MWD in stroke patients. We summarized the following four advantages of HIIT. First, HIIT can improve the cardiopulmonary function of stroke patients by increasing the stroke volume, myocardial contractility, and the number and volume of mitochondria in cells (63). Second, the exercise intensity of HIIT is high enough to stimulate both aerobic and anaerobic metabolic areas, so that the plasma volume and red blood cell volume are increased, and the venous return is improved. Ultimately, the stroke volume of the subjects increased (64, 65), the blood flow resistance decreased (66), the cardiopulmonary function was improved, and the effect was sustainable (67). Third, decreased neuromuscular recruitment after stroke can lead to decreased skeletal muscle oxidative capacity. HIIT can increase neuromuscular recruitment, reduce the proportion of fast-twitch (type II) muscle fibers, and change the corresponding aerobic oxidation substrates to increase aerobic endurance. Therefore, HIIT can reduce exercise fatigue in stroke patients (68, 69). Fourth, HIIT has significant advantages in terms of time efficiency. Meanwhile, the personalization of HIIT may enhance the enjoyment of exercise, thereby enhancing patients’ adherence to exercise (70).

Although HIIT is a highly effective exercise modality, the optimal exercise intensity, frequency, and duration are still controversial. Crozier et al. (69) recommended that the duration of high-intensity exercise should range from 30 s to 4 min, the interval recovery phase should range from 30 s to 3 min, and the total duration of a single intervention should be 25 to 30 min, which provides a valuable reference for the formulation of individualized plans for HIIT. Meanwhile, the recovery time after the end of HIIT is also critical. Because evidence suggests that older adults (mean age, 63.0 ± 3.4 years) train at least 3 days intervals to reduce the risk of fatigue and achieve optimal recovery (71). Therefore, HIIT can be performed twice a week at the beginning of training and then gradually increase the frequency of exercise as tolerated. In clinical practice, clinicians or rehabilitation therapists have the flexibility to formulate the best exercise prescription according to the specific condition of patients and the above-recommended programs.

In addition, whether HIIT increases the risk of acute cardiovascular events is still uncertain. Rognmo et al. (72) found the following in their analysis of 4,846 patients with coronary artery disease who underwent HIIT and MICT, “Of the total exercise time of 175,820 h, one fatal cardiac arrest was reported during MICT (129,456 h of exercise), and two nonfatal cardiac arrests were reported during HIIT (46,364 h of exercise), with no myocardial infarction.” The results showed that the risk of cardiovascular events was low for both types of exercise, but HIIT produced significant cardiovascular adaptations. Wewege et al. (73) conducted a systematic review study on the safety of HIIT in patients with cardiovascular disease, which included 23 studies involving 1,117 patients. Among the 23 studies, 14 used the classical 4 × 4 min long interval protocol for HIIT, and the rest lasted from 30 s to 3 min. The systematic review reported one adverse event for every 3,417 HIIT sessions (2,227 training hours); one adverse event occurred every 7,134 MICT sessions (5,606 training hours). There was no difference in the risk of adverse events between the two exercise methods. We, therefore, conclude that HIIT can be used as an additional option to traditional aerobic exercise to improve cardiopulmonary function in stroke patients with stable clinical symptoms, recent regular exercise, correct exercise risk screening before exercise intervention, and motor function monitoring during exercise. Finally, it should be noted that few patients with severe stroke or more comorbidities were included in these studies. Therefore, the existing research results cannot be directly generalized to all stroke patients, and cardiopulmonary rehabilitation programs for stroke patients with severe or more comorbidities should be further explored in the future.

We also assessed two indirect measures of cardiopulmonary function (SBP and DBP). Hypertension is the most important modifiable risk factor for stroke, and about 64% of stroke patients have a history of hypertension before onset (74). When SBP is >115 mmHg or DBP >75 mmHg, the likelihood of cardiovascular events increases with blood pressure (75). The risk of fatal cardiovascular events doubles with each increase in SBP (20 mmHg) or DBP (10 mmHg) (76). Therefore, optimizing the management of blood pressure is of great significance to improve the prognosis of stroke. Taking antihypertensive drugs to rapidly lower blood pressure, even to lower levels within the hypertensive range, can affect patients to varying degrees. Therefore, blood pressure management through exercise is undoubtedly a suitable rehabilitation method for stroke patients. CE is known internationally as “concurrent training” or “concurrent strength and endurance training” (77). Wilson et al. (78) believe that concurrent training is a method to obtain strength, muscle hypertrophy, and muscle endurance in the same training phase. Davis et al. (79) suggest that concurrent training can maintain physical strength levels, improve endurance and other essential physical qualities, and be more beneficial than traditional rehabilitation training. This NMA showed that CE was the most effective in improving SBP and DBP in stroke patients, and HIIT and AT also improved blood pressure in stroke patients to varying degrees. However, there was no significant difference in the effect of each intervention on blood pressure improvement (*p* > 0.05). Although exercise positively affects blood pressure and health status in patients with hypertension, excessively vigorous exercise in the short term may increase the risk of adverse events (80). To safely and effectively improve blood pressure in stroke patients, CE with moderate intensity (AT: 40–60% HRR, RT: 50–70% 1RM), 3 days per week for 20 weeks should be prioritized, as this exercise program has the best intervention effect (81). However, it should be noted that managing blood pressure in stroke patients is complicated due to the variability of the etiology and hemodynamics caused by stroke. Therefore, when exercise is used to reduce the blood pressure of stroke patients in clinical treatment, it should be combined with the specific condition of the patient, and the blood pressure should be controlled reasonably after a comprehensive evaluation.

Our study has some strengths. Firstly, 35 articles and 2,008 adult stroke survivors were included, indicating a large sample size. Secondly, the interventions included four methods of exercise and CT, and the effects of the interventions were evaluated by four outcome measures. Then, to ensure a good level of evidence, we strictly followed inclusion and exclusion criteria to ensure that only RCTs were included. Finally, our study is the first NMA to compare the effects of different exercise methods on improving cardiopulmonary function in stroke patients, providing a preliminary basis for further detailed research in this area. Our study also has some limitations that should be considered. First, the intensity, duration, frequency, and period of exercise interventions in the included studies were not consistent, and the types of exercise were also different, including treadmill exercise, power cycling exercise, and weight-bearing walking exercise, which may limit the results of the study. Second, the ages of the included patients are slightly different, and some data indicators will be affected by age, which will affect the quality of the article. Further subgroup analysis based on age is needed in the future. Third, the number of articles using RT as an intervention is small. At the same time, the limited amount of articles on improving SBP and DBP may reduce the reliability of the conclusions. Fourth, some studies did not describe random sequence generation and allocation concealment, which may cause certain selection biases. Finally, adverse events may not be strictly reported in the included RCTs. Therefore, the safety of exercise intervention needs to be further studied.

## 5 Conclusion

In this NMA, no single exercise method was optimal for all outcome indicators. Different exercise methods have distinct advantages in improving cardiopulmonary function in stroke patients. HIIT was more effective than other exercise methods in improving VO_2peak_ and 6MWD. CE was the most effective in improving DBP and SBP. Still, it is worth noting that the number of articles included in the latter two outcome measures is limited, and the conclusions still need to be further verified. At the same time, due to the limitations of existing clinical studies and evidence, larger sample size, multi-center, and high-quality RCTs are needed to verify the above conclusions in the future.

## Data availability statement

The original contributions presented in the study are included in the article/Supplementary material, further inquiries can be directed to the corresponding authors.

## Author contributions

CW: Writing – original draft. YX: Writing – original draft. LZ: Data curation, Software, Writing – review & editing. WF: Data curation, Software, Writing – review & editing. ZL: Data curation, Software, Writing – review & editing. MY: Methodology, Writing – review & editing. LW: Project administration, Supervision, Writing – review & editing. All authors contributed to the article and approved the submitted version.
