# Supplementary material for: Comparative efficacy of different exercise methods to improve cardiopulmonary function in stroke patients: a network meta-analysis of randomized controlled trials
Source: Front Neurol. 2024 Jan 17;15:1288032. doi: 10.3389/fneur.2024.1288032 (PMC10836840; doi:10.3389/fneur.2024.1288032)
Supplement: Supplementary file 1 [file Data_Sheet_1.PDF]

Supplementary Material

1. Supplementary Figures and Tables

1.2 Supplementary Figures

	HIIT			CT				Mean Difference
Study or Subgroup	Mean	SD	Total	Mean	SD	Total	Weight	IV, Fixed, 95% CI
Gjellesvik et al., 2020	3.05	10.88	33	-1.59	8.04	31	1.3%	4.64 [-0.03, 9.31]
Ye et al., 2020	4.63	1.68	60	1.03	1.31	60	98.7%	3.60 [3.06, 4.14]
**Total (95% CI)**			**93**			**91**	**100.0%**	**3.61 [3.08, 4.15]**

Heterogeneity: Chi² = 0.19, df = 1 (P = 0.66); I² = 0%
Test for overall effect: Z = 13.23 (P < 0.00001)

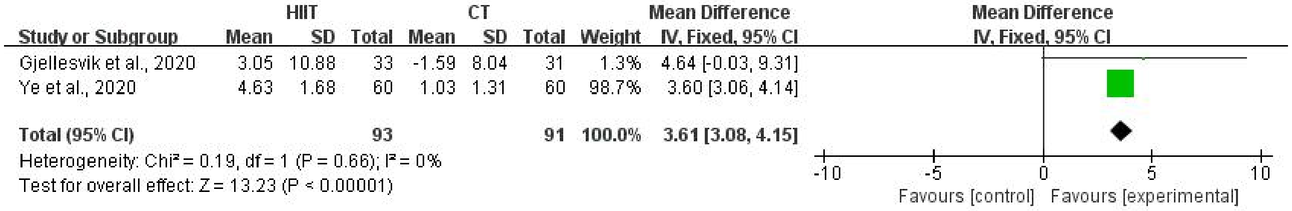

	AT			CT				Mean Difference
Study or Subgroup	Mean	SD	Total	Mean	SD	Total	Weight	IV, Random, 95% CI
Chang et al., 2012	2.3	5.65	20	1.3	4.9	17	3.0%	1.00 [-2.40, 4.40]
Globas et al., 2012	5.5	5.9	18	0.8	7.8	18	1.9%	4.70 [0.18, 9.22]
Gu et al., 2019	3.1	4.75	43	1.3	5.03	43	5.7%	1.80 [-0.27, 3.87]
Han et al., 2022	2.73	1.88	69	1.32	1.9	69	11.1%	1.41 [0.78, 2.04]
Jin et al., 2013	3.6	1.4	65	0.1	1	63	11.7%	3.50 [3.08, 3.92]
Lee et al., 2008	1.5	4.23	14	-0.8	3.96	12	3.4%	2.30 [-0.85, 5.45]
Lennon et al., 2008	1.4	1.98	23	0	1.85	23	9.3%	1.40 [0.29, 2.51]
Mackay-Lyons et al., 2013	4.2	4.16	22	1.1	3.9	23	5.0%	3.10 [0.74, 5.46]
Macko et al., 2005	2.4	0.95	25	0.2	1	20	11.3%	2.20 [1.62, 2.78]
Rimmer et al., 2009	0.65	7.5	14	-0.35	3.93	13	1.9%	1.00 [-3.47, 5.47]
Severinsen et al., 2014	2.5	2.43	13	0.06	2.24	16	6.9%	2.44 [0.72, 4.16]
Stoller et al., 2015	3.2	5.66	7	3.2	5.76	7	1.2%	0.00 [-5.98, 5.98]
Tang et al., 2009	1.5	3.93	23	0.9	3.75	22	5.2%	0.60 [-1.64, 2.84]
Tang et al., 2014	0.5	7.05	22	-0.3	5.74	25	2.6%	0.80 [-2.91, 4.51]
Vanroy et al., 2017	2.94	4.93	31	1.12	4.93	25	4.4%	1.82 [-0.78, 4.42]
Xu et al., 2017	3	3.61	13	0	4	13	3.7%	3.00 [0.07, 5.93]
Ye et al., 2020	2.2	1.15	60	1.03	1.31	60	11.7%	1.17 [0.73, 1.61]
**Total (95% CI)**			**482**			**469**	**100.0%**	**1.97 [1.29, 2.65]**

Heterogeneity: Tau² = 0.98; Chi² = 71.63, df = 16 (P < 0.00001); I² = 78%
Test for overall effect: Z = 5.67 (P < 0.00001)

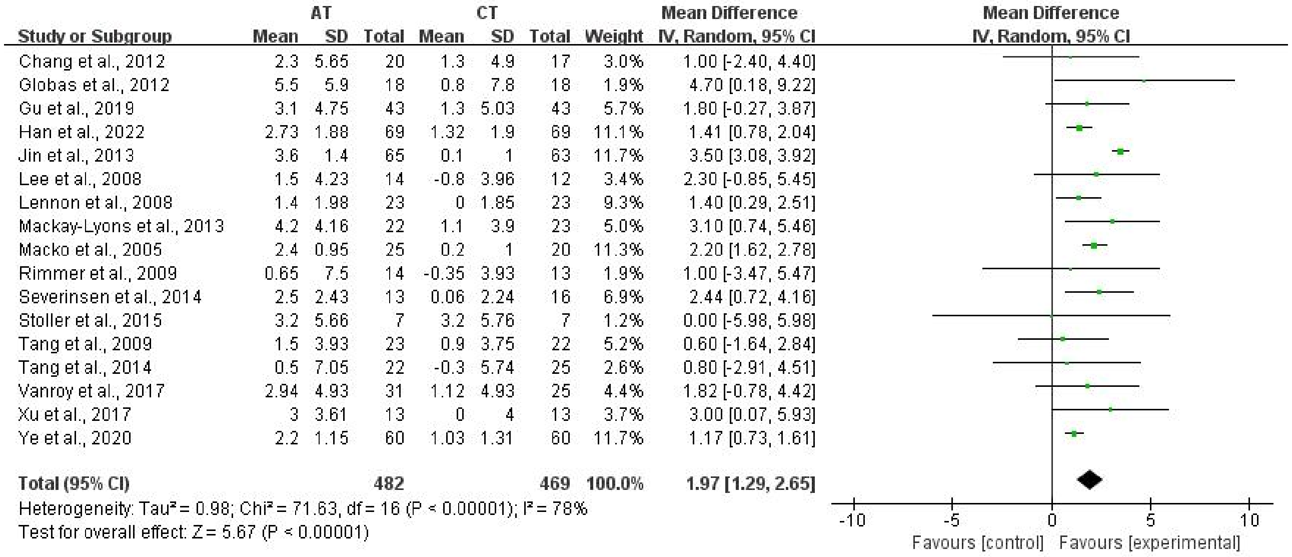

	RT			CT				Mean Difference
Study or Subgroup	Mean	SD	Total	Mean	SD	Total	Weight	IV, Random, 95% CI
Ivey et al., 2017	1.2	1.55	22	-0.4	1.1	16	37.9%	1.60 [0.76, 2.44]
Kang et al., 2022	4.54	5.82	15	-1.35	3.26	15	14.5%	5.89 [2.51, 9.27]
Lee et al., 2008	-0.5	3.58	13	-0.8	3.96	12	17.0%	0.30 [-2.67, 3.27]
Severinsen et al., 2014	0.6	2.08	14	0.06	2.24	17	30.5%	0.54 [-0.98, 2.06]
**Total (95% CI)**			**64**			**60**	**100.0%**	**1.68 [0.09, 3.26]**

Heterogeneity: Tau² = 1.53; Chi² = 8.70, df = 3 (P = 0.03); I² = 66%
Test for overall effect: Z = 2.08 (P = 0.04)

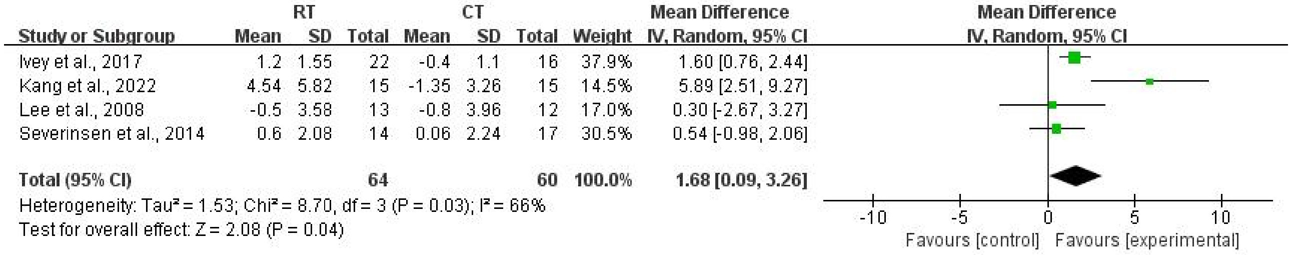

	CE			CT				Mean Difference
Study or Subgroup	Mean	SD	Total	Mean	SD	Total	Weight	IV, Random, 95% CI
Duncan et al., 2003	1.05	1.63	50	0.06	1.63	50	41.9%	0.99 [0.35, 1.63]
Kang et al., 2022	5.11	6.09	15	-1.35	3.26	15	28.6%	6.46 [2.96, 9.96]
Lee et al., 2008	2.2	4.53	13	-0.8	3.96	12	29.5%	3.00 [-0.33, 6.33]
**Total (95% CI)**			**78**			**77**	**100.0%**	**3.15 [-0.12, 6.42]**

Heterogeneity: Tau² = 6.55; Chi² = 10.23, df = 2 (P = 0.006); I² = 80%
Test for overall effect: Z = 1.89 (P = 0.06)

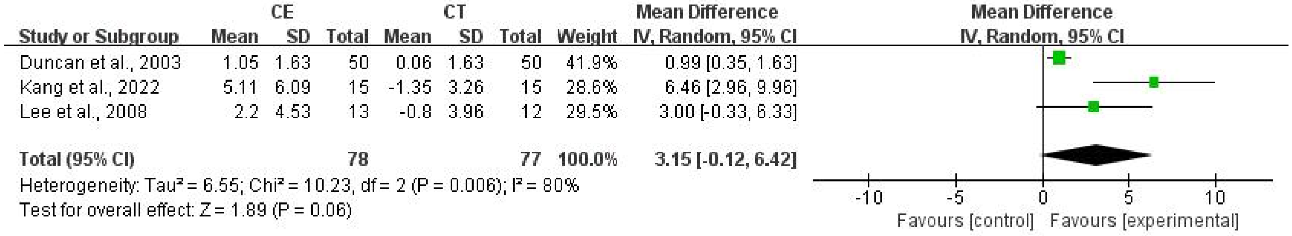

Study or Subgroup	HIIT Mean	HIIT SD	HIIT Total	AT Mean	AT SD	AT Total	Weight	Mean Difference IV, Random, 95% CI
Boyne et al., 2016	2.2	4.6	11	-1.3	6.8	5	3.7%	3.50 [-3.05, 10.05]
Hsu et al., 2020	3.4	3.74	10	1.7	6.11	13	8.2%	1.70 [-2.35, 5.75]
Ivey et al., 2015	5.4	1.65	18	0.9	1.15	16	28.0%	4.50 [3.55, 5.45]
Munari et al., 2018	4.6	4.78	8	0.86	4.83	7	6.1%	3.74 [-1.14, 8.62]
Soh et al., 2020	4.7	2.15	18	0.1	2.43	18	23.1%	4.60 [3.10, 6.10]
Ye et al., 2020	4.63	1.68	60	2.2	1.15	60	31.0%	2.43 [1.91, 2.95]
**Total (95% CI)**			**125**			**119**	**100.0%**	**3.57 [2.23, 4.91]**

Heterogeneity: Tau² = 1.43; Chi² = 19.18, df = 5 (P = 0.002); I² = 74%
Test for overall effect: Z = 5.24 (P < 0.00001)

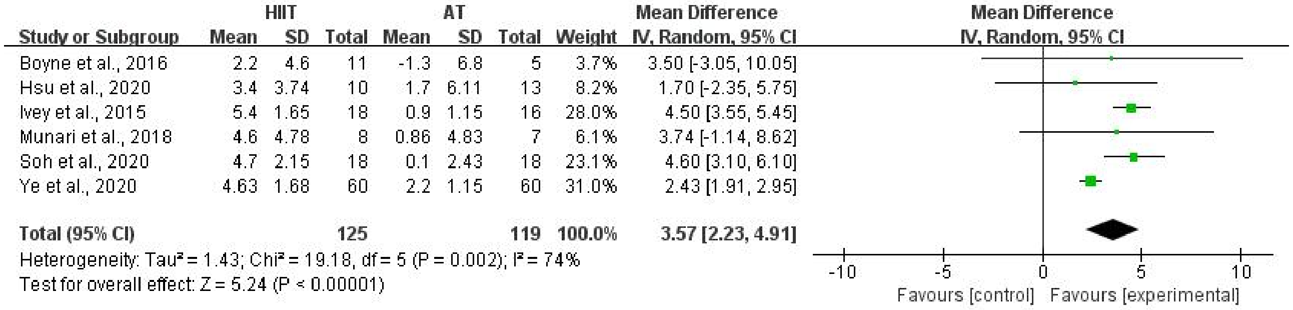

Study or Subgroup	AT Mean	AT SD	AT Total	RT Mean	RT SD	RT Total	Weight	Mean Difference IV, Fixed, 95% CI
Lee et al., 2008	1.5	4.23	12	-0.5	3.58	12	23.0%	2.00 [-1.14, 5.14]
Severinsen et al., 2014	2.5	2.43	13	0.6	2.08	14	77.0%	1.90 [0.19, 3.61]
**Total (95% CI)**			**25**			**26**	**100.0%**	**1.92 [0.42, 3.43]**

Heterogeneity: Chi² = 0.00, df = 1 (P = 0.96); I² = 0%
Test for overall effect: Z = 2.51 (P = 0.01)

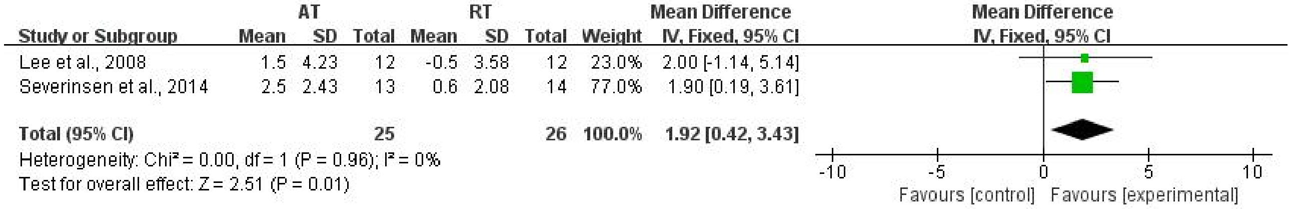

Study or Subgroup	RT Mean	RT SD	RT Total	CE Mean	CE SD	CE Total	Weight	Mean Difference IV, Fixed, 95% CI
Kang et al., 2022	4.54	5.82	7	5.11	6.09	11	25.3%	-0.57 [-6.19, 5.05]
Lee et al., 2008	-0.5	3.58	12	2.2	4.53	12	74.7%	-2.70 [-5.97, 0.57]
**Total (95% CI)**			**19**			**23**	**100.0%**	**-2.16 [-4.99, 0.66]**

Heterogeneity: Chi² = 0.41, df = 1 (P = 0.52); I² = 0%
Test for overall effect: Z = 1.50 (P = 0.13)

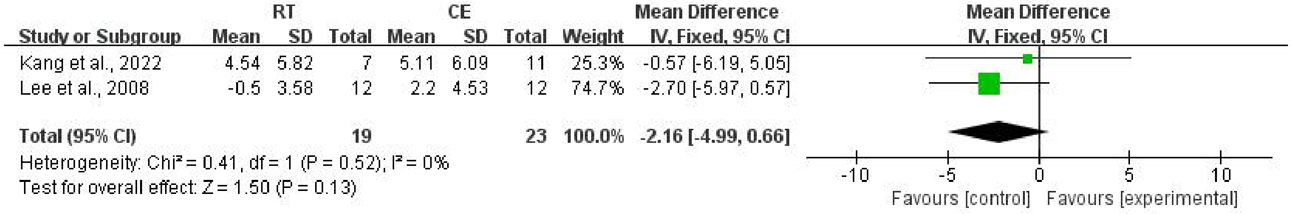

Study or Subgroup	AT Mean	AT SD	AT Total	CE Mean	CE SD	CE Total	Weight	Mean Difference IV, Fixed, 95% CI
Carr et al., 2003	0.4	3.86	20	2.8	5.37	20	19.6%	-2.40 [-5.30, 0.50]
Lee et al., 2008	1.5	4.23	12	2.2	4.53	12	13.4%	-0.70 [-4.21, 2.81]
Marzolini et al., 2018	1.7	2.7	35	1.9	4.9	33	45.9%	-0.20 [-2.10, 1.70]
Ran et al., 2022	3.32	2.91	20	5.53	5.69	20	21.0%	-2.21 [-5.01, 0.59]
**Total (95% CI)**			**87**			**85**	**100.0%**	**-1.12 [-2.41, 0.16]**

Heterogeneity: Chi² = 2.29, df = 3 (P = 0.51); I² = 0%
Test for overall effect: Z = 1.71 (P = 0.09)

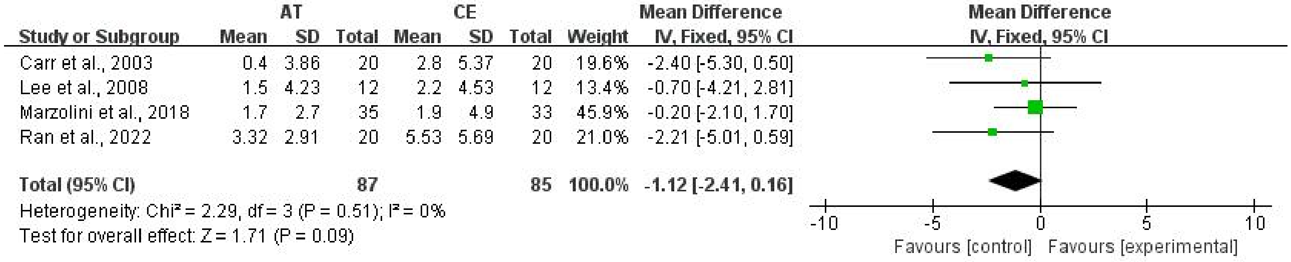

Supplementary Figure 1. Forest plots of the pairwise meta-analysis for VO_{2peak}. HIIT, high-intensity interval training; AT, aerobic training; RT, resistance training; CE, combined aerobic and resistance exercise; CT, conventional therapy.

Study or Subgroup	HIIT Mean	HIIT SD	HIIT Total	CT Mean	CT SD	CT Total	Weight	Mean Difference IV, Fixed, 95% CI
Gjelleskvik et al., 2021	39.7	155.2	33	-8.1	135.8	31	34.8%	47.80 [-23.54, 119.14]
Sandberg et al., 2016	105.1	79.5	29	35.9	115.1	27	65.2%	69.20 [17.03, 121.37]
**Total (95% CI)**			**62**			**58**	**100.0%**	**61.74 [19.63, 103.86]**

Heterogeneity: Chi² = 0.23, df = 1 (P = 0.64); I² = 0%
Test for overall effect: Z = 2.87 (P = 0.004)

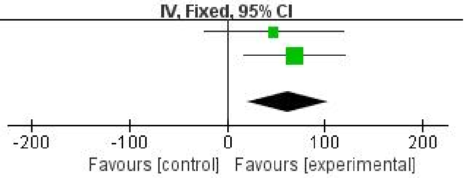

Study or Subgroup	AT Mean	AT SD	AT Total	CT Mean	CT SD	CT Total	Weight	Mean Difference IV, Random, 95% CI
Bang et al., 2016	66.7	47.9	6	29.5	47.2	6	11.7%	37.20 [-16.61, 91.01]
Globas et al., 2012	57.7	127.4	18	4.7	183.3	18	5.8%	53.00 [-50.12, 156.12]
Han et al., 2022	209.13	52.22	69	161.3	52.21	69	17.6%	47.83 [30.41, 65.25]
Jin et al., 2013	6.9	64.3	65	1.3	51.4	63	17.3%	5.60 [-14.54, 25.74]
Lee et al., 2008	12.2	160.55	12	4.9	162.1	12	4.2%	7.30 [-121.79, 136.39]
Mackay-Lyons et al., 2013	12.2	160.55	12	4.9	162.1	12	4.2%	7.30 [-121.79, 136.39]
Macko et al., 2005	161	76.18	25	20	104.79	20	11.5%	141.00 [86.22, 195.78]
Severinsen et al., 2014	19	53.49	13	43.5	47.65	16	14.5%	-24.50 [-61.79, 12.79]
Tang et al., 2009	127.2	206.52	23	89.5	185.58	22	5.0%	37.70 [-76.92, 152.32]
Tang et al., 2014	19.9	131.44	22	9.3	145.92	25	8.1%	10.60 [-68.70, 89.90]
**Total (95% CI)**			**265**			**263**	**100.0%**	**32.90 [2.89, 62.91]**

Heterogeneity: Tau² = 1251.86; Chi² = 34.38, df = 9 (P < 0.0001); I² = 74%
Test for overall effect: Z = 2.15 (P = 0.03)

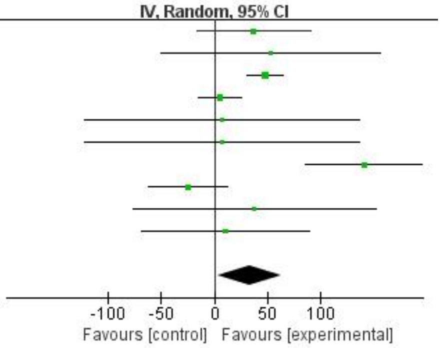

Study or Subgroup	RT Mean	RT SD	RT Total	CT Mean	CT SD	CT Total	Weight	Mean Difference IV, Random, 95% CI
Fu et al.,2022	62.51	50.87	20	28.89	43.08	20	25.7%	33.62 [4.41, 62.83]
Ivey et al., 2017	132	93.05	14	-3	111.05	16	13.2%	135.00 [61.95, 208.05]
Lee et al., 2008	7.4	145.06	12	4.9	162.1	12	6.4%	2.50 [-120.58, 125.58]
Severinsen et al., 2014	29.6	45.9	14	43.5	47.65	16	24.3%	-13.90 [-47.41, 19.61]
Shao et al., 2023	137.82	27.51	64	88.65	36.31	63	30.4%	49.17 [37.95, 60.39]
**Total (95% CI)**			**124**			**127**	**100.0%**	**38.19 [3.28, 73.10]**

Heterogeneity: Tau² = 1012.13; Chi² = 19.14, df = 4 (P = 0.0007); I² = 79%
Test for overall effect: Z = 2.14 (P = 0.03)

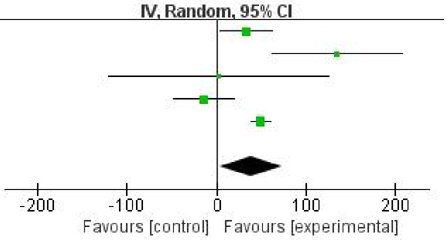

Study or Subgroup	CE Mean	CE SD	CE Total	CT Mean	CT SD	CT Total	Weight	Mean Difference IV, Fixed, 95% CI
Duncan et al., 2003	61.61	70.5	44	33.59	51.83	48	91.4%	28.02 [2.55, 53.49]
Lee et al., 2008	24.2	130.31	12	4.9	162.1	12	4.3%	19.30 [-98.38, 136.98]
Toledano-Zarhi A et al., 2011	53.3	181.6	13	24.9	119.6	14	4.3%	28.40 [-88.52, 145.32]
**Total (95% CI)**			**69**			**74**	**100.0%**	**27.66 [3.31, 52.01]**

Heterogeneity: Chi² = 0.02, df = 2 (P = 0.99); I² = 0%
Test for overall effect: Z = 2.23 (P = 0.03)

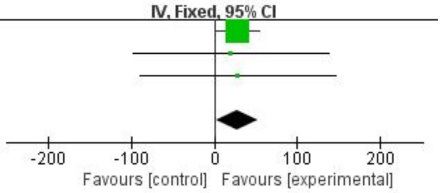

Study or Subgroup	HIIT Mean	HIIT SD	HIIT Total	AT Mean	AT SD	AT Total	Weight	Mean Difference IV, Fixed, 95% CI
Boyne et al., 2016	15	28	11	15	51	5	59.8%	0.00 [-47.67, 47.67]
Ivey et al., 2015	184	120.13	18	104	74.55	16	30.8%	80.00 [13.56, 146.44]
Munari et al., 2018	64.25	118.46	8	6.1	119.13	7	9.4%	58.15 [-62.38, 178.68]
**Total (95% CI)**			**37**			**28**	**100.0%**	**30.08 [-6.79, 66.96]**

Heterogeneity: Chi² = 3.91, df = 2 (P = 0.14); I² = 49%
Test for overall effect: Z = 1.60 (P = 0.11)

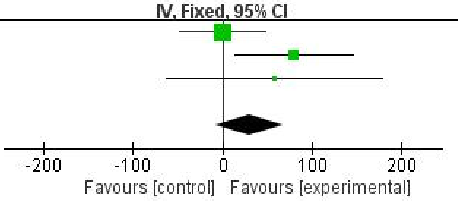

Study or Subgroup	AT Mean	AT SD	AT Total	RT Mean	RT SD	RT Total	Weight	Mean Difference IV, Fixed, 95% CI
Lee et al., 2008	12.2	160.55	12	7.4	145.06	12	8.7%	4.80 [-117.62, 127.22]
Severinsen et al., 2014	19	53.49	13	29.6	45.9	14	91.3%	-10.60 [-48.33, 27.13]
**Total (95% CI)**			**25**			**26**	**100.0%**	**-9.26 [-45.32, 26.79]**

Heterogeneity: Chi² = 0.06, df = 1 (P = 0.81); I² = 0%
Test for overall effect: Z = 0.50 (P = 0.61)

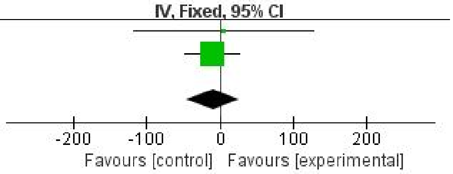

Study or Subgroup	AT Mean	AT SD	AT Total	CE Mean	CE SD	CE Total	Weight	Mean Difference IV, Fixed, 95% CI
Lee et al., 2008	12.2	160.55	12	24.2	130.31	12	5.6%	-12.00 [-128.99, 104.99]
Marzolini et al., 2018	36.5	63.7	35	39.9	55.6	33	94.4%	-3.40 [-31.78, 24.98]
**Total (95% CI)**			**47**			**45**	**100.0%**	**-3.88 [-31.45, 23.70]**

Heterogeneity: Chi² = 0.02, df = 1 (P = 0.89); I² = 0%
Test for overall effect: Z = 0.28 (P = 0.78)

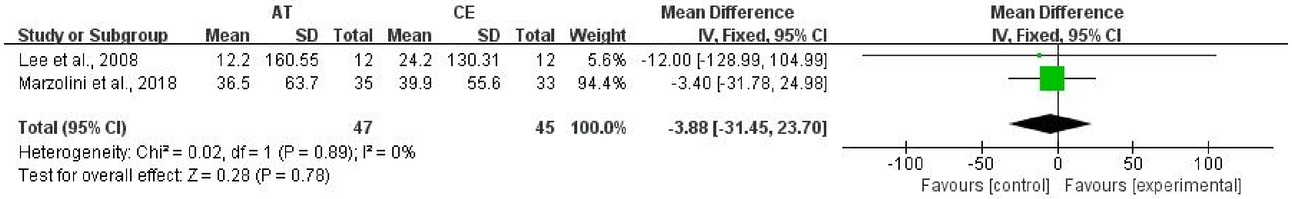

Study or Subgroup	RT Mean	RT SD	RT Total	CE Mean	CE SD	CE Total	Weight	Mean Difference IV, Fixed, 95% CI
Lee et al., 2008	7.4	145.06	12	24.2	130.31	12	100.0%	-16.80 [-127.13, 93.53]
**Total (95% CI)**			**12**			**12**	**100.0%**	**-16.80 [-127.13, 93.53]**

Heterogeneity: Not applicable
Test for overall effect: Z = 0.30 (P = 0.77)

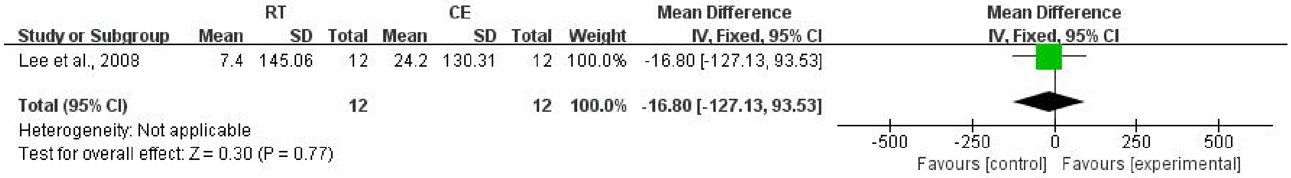

Supplementary Figure 2. Forest plots of the pairwise meta-analysis for 6MWD. HIIT, high-intensity interval training; AT, aerobic training; RT, resistance training; CE, combined aerobic and resistance exercise; CT, conventional therapy.

Study or Subgroup	HIIT Mean	HIIT SD	HIIT Total	CT Mean	CT SD	CT Total	Weight	Mean Difference IV, Fixed, 95% CI
Gjellesvik et al., 2020	-2.76	14.95	33	-2.84	14.28	31	100.0%	0.08 [-7.08, 7.24]
**Total (95% CI)**			**33**			**31**	**100.0%**	**0.08 [-7.08, 7.24]**
Heterogeneity: Not applicable								
Test for overall effect: Z = 0.02 (P = 0.98)								

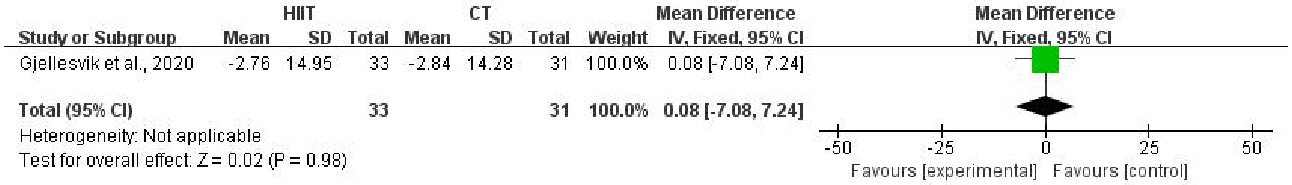

Study or Subgroup	AT Mean	AT SD	AT Total	CT Mean	CT SD	CT Total	Weight	Mean Difference IV, Fixed, 95% CI
Jin et al., 2013	-1	15.02	65	-0.8	15.39	63	67.6%	-0.20 [-5.47, 5.07]
Lennon et al., 2008	-1.1	14.65	23	-2.7	17.11	23	22.1%	1.60 [-7.61, 10.81]
Rimmer et al., 2009	-10.3	19.22	14	-2.6	16.55	13	10.3%	-7.70 [-21.20, 5.80]
**Total (95% CI)**			**102**			**99**	**100.0%**	**-0.57 [-4.91, 3.76]**
Heterogeneity: Chi² = 1.30, df = 2 (P = 0.52); I² = 0%								
Test for overall effect: Z = 0.26 (P = 0.80)								

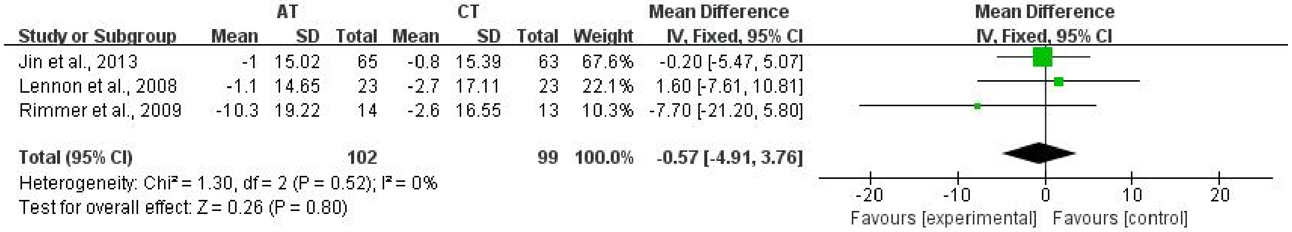

Study or Subgroup	CE Mean	CE SD	CE Total	CT Mean	CT SD	CT Total	Weight	Mean Difference IV, Fixed, 95% CI
Toledano-Zarhi A et al., 2011	-3.2	16.65	13	0	15.34	14	100.0%	-3.20 [-15.30, 8.90]
**Total (95% CI)**			**13**			**14**	**100.0%**	**-3.20 [-15.30, 8.90]**
Heterogeneity: Not applicable								
Test for overall effect: Z = 0.52 (P = 0.60)								

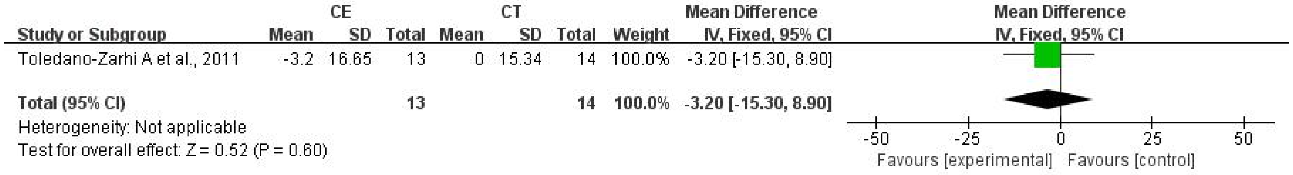

Study or Subgroup	HIIT Mean	HIIT SD	HIIT Total	AT Mean	AT SD	AT Total	Weight	Mean Difference IV, Fixed, 95% CI
Soh et al., 2020	1.2	8.43	18	2.5	7.76	18	100.0%	-1.30 [-6.59, 3.99]
**Total (95% CI)**			**18**			**18**	**100.0%**	**-1.30 [-6.59, 3.99]**
Heterogeneity: Not applicable								
Test for overall effect: Z = 0.48 (P = 0.63)								

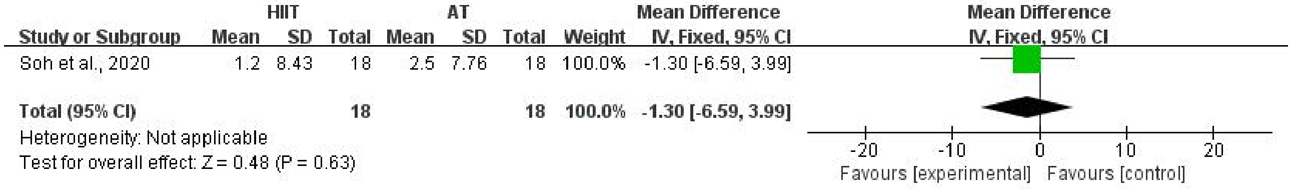

Study or Subgroup	AT Mean	AT SD	AT Total	CT Mean	CT SD	CT Total	Weight	Mean Difference IV, Fixed, 95% CI
Jin et al., 2013	-1	15.02	65	-0.8	15.39	63	67.6%	-0.20 [-5.47, 5.07]
Lennon et al., 2008	-1.1	14.65	23	-2.7	17.11	23	22.1%	1.60 [-7.61, 10.81]
Rimmer et al., 2009	-10.3	19.22	14	-2.6	16.55	13	10.3%	-7.70 [-21.20, 5.80]
**Total (95% CI)**			**102**			**99**	**100.0%**	**-0.57 [-4.91, 3.76]**
Heterogeneity: Chi² = 1.30, df = 2 (P = 0.52); I² = 0%								
Test for overall effect: Z = 0.26 (P = 0.80)								

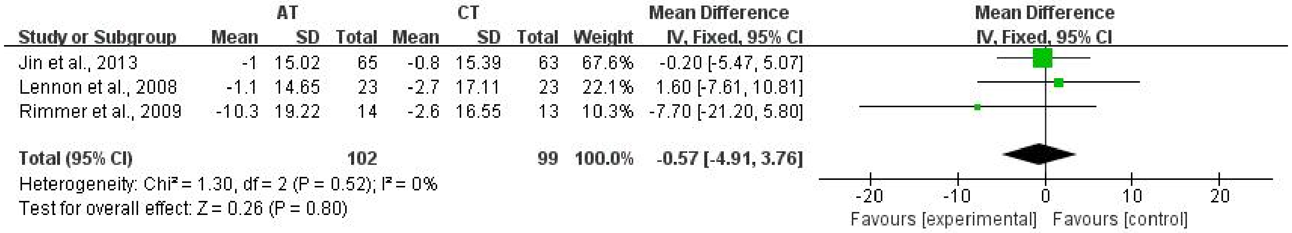

Supplementary Figure 3. Forest plots of the pairwise meta-analysis for SBP. HIIT, high-intensity interval training; AT, aerobic training; RT, resistance training; CE, combined aerobic and resistance exercise; CT, conventional therapy.

Study or Subgroup	HIIT Mean	HIIT SD	HIIT Total	CT Mean	CT SD	CT Total	Weight	Mean Difference IV, Fixed, 95% CI
Gjellesvik et al., 2020	-0.28	10.15	33	-0.56	9.11	31	100.0%	0.28 [-4.44, 5.00]
**Total (95% CI)**			**33**			**31**	**100.0%**	**0.28 [-4.44, 5.00]**

Heterogeneity: Not applicable
Test for overall effect: Z = 0.12 (P = 0.91)

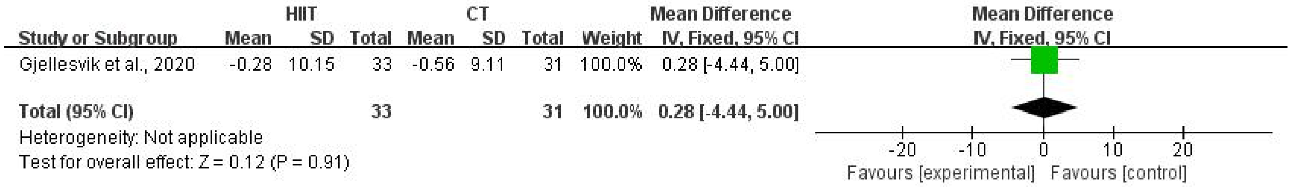

Study or Subgroup	AT Mean	AT SD	AT Total	CT Mean	CT SD	CT Total	Weight	Mean Difference IV, Fixed, 95% CI
Jin et al., 2013	-0.8	10.73	65	0.8	10.86	63	59.5%	-1.60 [-5.34, 2.14]
Lennon et al., 2008	1.7	8.03	23	0.2	10.54	23	28.4%	1.50 [-3.92, 6.92]
Rimmer et al., 2009	-8.7	11.62	14	-0.5	10.42	13	12.1%	-8.20 [-16.51, 0.11]
**Total (95% CI)**			**102**			**99**	**100.0%**	**-1.51 [-4.40, 1.37]**

Heterogeneity: $Chi^2 = 3.68$, df = 2 (P = 0.16); $I^2 = 46\%$
Test for overall effect: Z = 1.03 (P = 0.30)

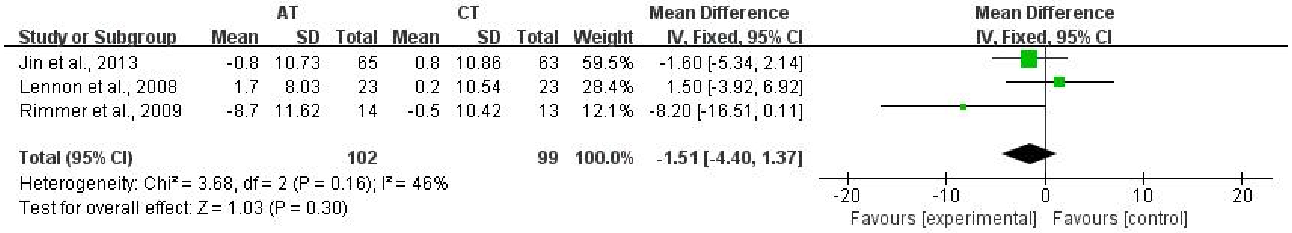

Study or Subgroup	CE Mean	CE SD	CE Total	CT Mean	CT SD	CT Total	Weight	Mean Difference IV, Fixed, 95% CI
Mackay-Lyons et al., 2013	-1.9	7.39	13	-1.8	6.82	14	100.0%	-0.10 [-5.48, 5.28]
**Total (95% CI)**			**13**			**14**	**100.0%**	**-0.10 [-5.48, 5.28]**

Heterogeneity: Not applicable
Test for overall effect: Z = 0.04 (P = 0.97)

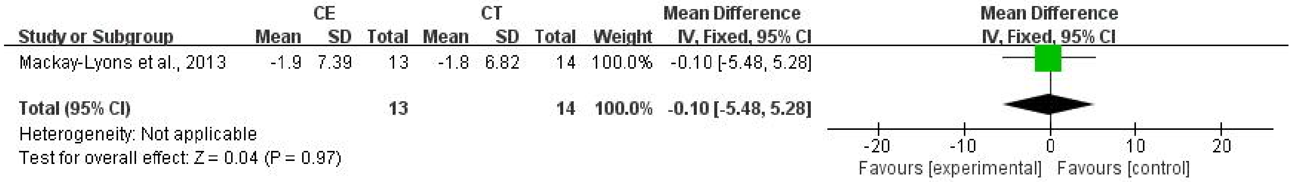

Study or Subgroup	HIIT Mean	HIIT SD	HIIT Total	AT Mean	AT SD	AT Total	Weight	Mean Difference IV, Fixed, 95% CI
Soh et al., 2020	2	7.57	18	2.4	7.84	18	100.0%	-0.40 [-5.43, 4.63]
**Total (95% CI)**			**18**			**18**	**100.0%**	**-0.40 [-5.43, 4.63]**

Heterogeneity: Not applicable
Test for overall effect: Z = 0.16 (P = 0.88)

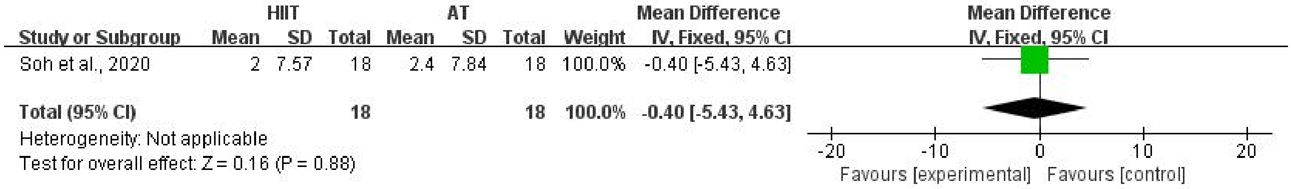

Study or Subgroup	AT Mean	AT SD	AT Total	CE Mean	CE SD	CE Total	Weight	Mean Difference IV, Fixed, 95% CI
Marzolini et al., 2018	0.8	8.2	35	-2.9	10	33	100.0%	3.70 [-0.66, 8.06]
**Total (95% CI)**			**35**			**33**	**100.0%**	**3.70 [-0.66, 8.06]**

Heterogeneity: Not applicable
Test for overall effect: Z = 1.66 (P = 0.10)

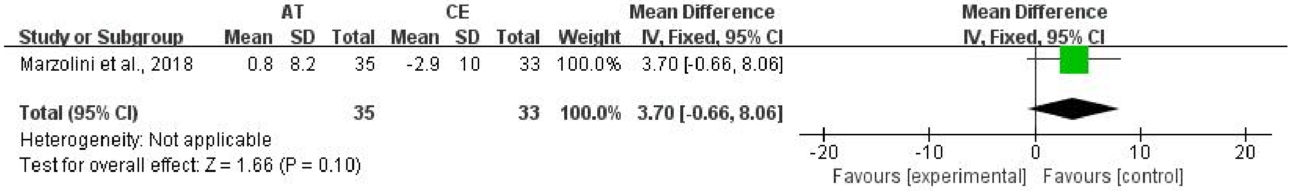

Supplementary Figure 4. Forest plots of the pairwise meta-analysis for DBP. HIIT, high-intensity interval training; AT, aerobic training; RT, resistance training; CE, combined aerobic and resistance exercise; CT, conventional therapy.

A

```
Estimated between-studies SDs and correlation matrix:
              SD          _y_B        _y_C        _y_D        _y_E
 _y_B  .72937488           1           .           .           .
 _y_C  .72937488          .5           1           .           .
 _y_D  .72937488          .5          .5           1           .
 _y_E  .72937488          .5          .5          .5           1

 Testing for inconsistency:
  ( 1)  [_y_B]des_ABE = 0
  ( 2)  [_y_E]des_AE = 0
  ( 3)  [_y_E]des_BCDE = 0
  ( 4)  [_y_E]des_BCE = 0
  ( 5)  [_y_C]des_BCE = 0
  ( 6)  [_y_D]des_BD = 0
  ( 7)  [_y_E]des_BE = 0
  ( 8)  [_y_C]des_CDE = 0
  ( 9)  [_y_D]des_CDE = 0
  (10)  [_y_C]des_CE = 0
  (11)  [_y_D]des_DE = 0

           chi2( 11) =   18.59
         Prob > chi2 =    0.0690
mvmeta command stored as F9; test command stored as F8
```

B

```
Estimated between-studies SDs and correlation matrix:
              SD          _y_B        _y_C        _y_D        _y_E
 _y_B  32.346276           1           .           .           .
 _y_C  32.346276          .5           1           .           .
 _y_D  32.346276          .5          .5           1           .
 _y_E  32.346276          .5          .5          .5           1

 Testing for inconsistency:
  ( 1)  [_y_E]des_BCDE = 0
  ( 2)  [_y_E]des_BCE = 0
  ( 3)  [_y_C]des_BCE = 0
  ( 4)  [_y_D]des_BD = 0
  ( 5)  [_y_E]des_BE = 0
  ( 6)  [_y_C]des_CE = 0
  ( 7)  [_y_D]des_DE = 0

           chi2(  7) =    5.01
         Prob > chi2 =    0.6592
mvmeta command stored as F9; test command stored as F8
```

C

```
Estimated between-studies SDs and correlation matrix:
              SD          _y_B        _y_C        _y_D
 _y_B  1.927e-08           1           .           .
 _y_C  1.927e-08          .5           1           .
 _y_D  1.927e-08          .5          .5           1

 Testing for inconsistency:
  ( 1)  [_y_D]des_BD = 0
  ( 2)  [_y_C]des_CD = 0

           chi2(  2) =    0.15
         Prob > chi2 =    0.9259
mvmeta command stored as F9; test command stored as F8
```

D

```
Estimated between-studies SDs and correlation matrix:
              SD          _y_B        _y_C        _y_D
 _y_B  2.1241019           1           .           .
 _y_C  2.1241019          .5           1           .
 _y_D  2.1241019          .5          .5           1

 Testing for inconsistency:
  ( 1)  [_y_D]des_BD = 0
  ( 2)  [_y_C]des_CD = 0

           chi2(  2) =    1.17
         Prob > chi2 =    0.5571
mvmeta command stored as F9; test command stored as F8
```

Supplementary Figure 5. Local inconsistency test. (A) VO_{2peak}; (B) 6WMD; (C) SBP; (D) DBP.

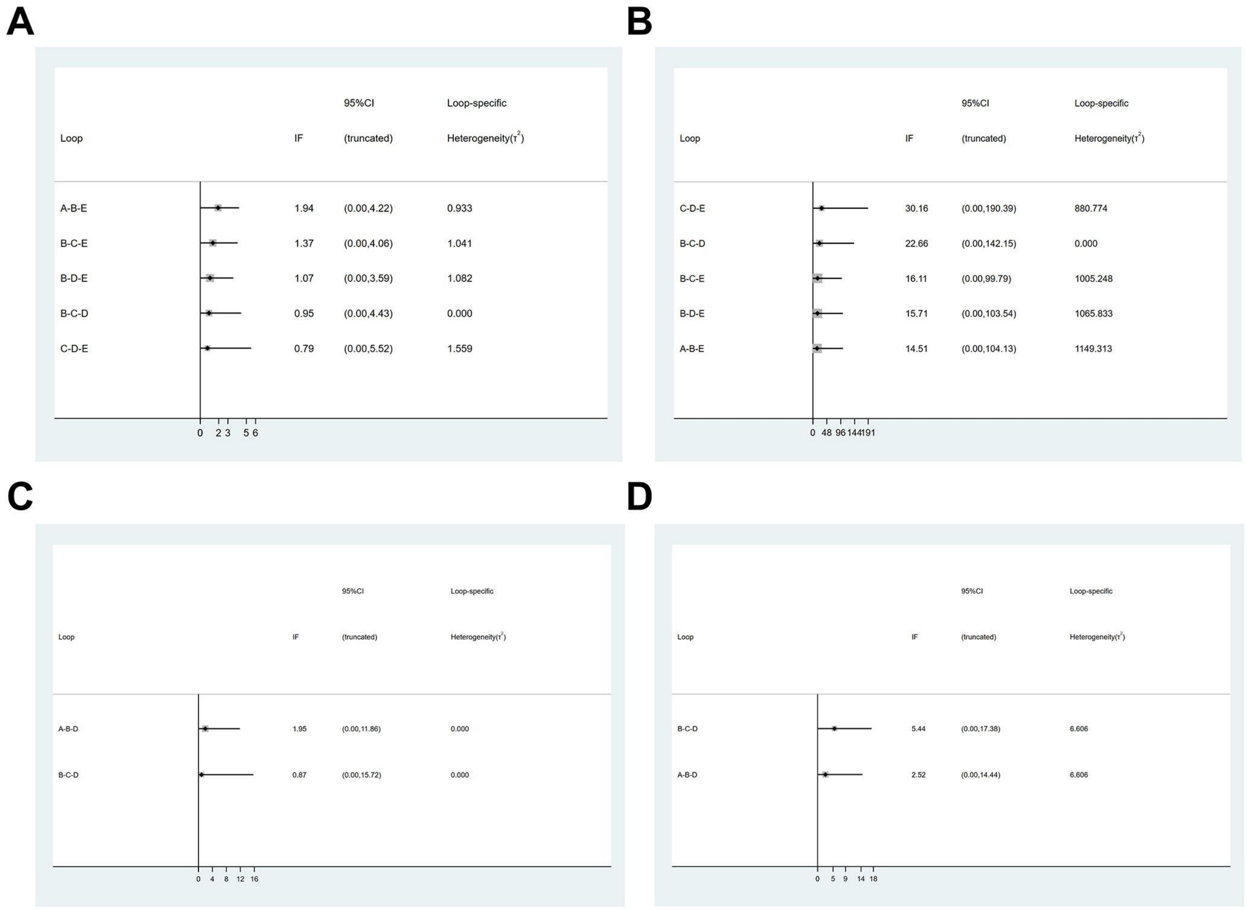

Supplementary Figure 6. Loop inconsistency test. (A) VO_{2peak}; (B) 6WMD; (C) SBP; (D) DBP.

A

Egger's test

Std_Eff	Coefficient	Std. err.	t	P>|t|	[95% conf. interval]	
slope	.7372091	.4393611	1.68	0.103	-.1577401	1.632158
bias	.326145	1.41352	0.23	0.819	-2.553102	3.205392

B

Egger's test

Std_Eff	Coefficient	Std. err.	t	P>|t|	[95% conf. interval]	
slope	.7784942	.3350724	2.32	0.030	.0835966	1.473392
bias	-1.016587	1.143804	-0.89	0.384	-3.388691	1.355518

C

Egger's test

Std_Eff	Coefficient	Std. err.	t	P>|t|	[95% conf. interval]	
slope	.1827165	.1960301	0.93	0.394	-.321195	.686628
bias	-1.050548	.7356774	-1.43	0.213	-2.941667	.8405712

D

Egger's test

Std_Eff	Coefficient	Std. err.	t	P>|t|	[95% conf. interval]	
slope	-.0565728	.3744508	-0.15	0.886	-1.019129	.9059838
bias	-.3511089	1.401019	-0.25	0.812	-3.952543	3.250325

Supplementary Figure 7. Egger's test. (A) VO_{2peak}; (B) 6WMD; (C) SBP; (D) DBP.

1.2 Supplementary Tables

Supplementary Table 1. Search strategy (using PubMed as an example).

Number	Search terms
#1	stroke [Mesh]
#2	apoplexy [Title/Abstract] OR hemiplegia [Title/Abstract] OR cerebrovascular disease [Title/Abstract] OR cerebral infarction [Title/Abstract] OR cerebral hemorrhage [Title/Abstract]
#3	#1 OR #2
#4	exercise [Title/Abstract] OR sport [Mesh] OR train [Title/Abstract] OR physical activity [Title/Abstract] OR resistance exercise [Title/Abstract] OR aerobic exercise [Title/Abstract] OR high-intensity interval training [Mesh]
#5	random [Title/Abstract] OR randomized controlled trial [Title/Abstract] OR RCT [Title/Abstract]
#6	#3 AND #4 AND #5

Supplementary Table 2. Local consistency test for VO_{2peak}.

Side	Direct		Indirect		Difference			tau
	Coef.	Std. Err.	Coef.	Std. Err.	Coef.	Std. Err.	P>z	
**HIIT vs AT**	-3.567652	.5630788	-1.27997	1.496067	-2.287682	1.593395	0.151	.916086
**HIIT vs CT**	-3.711766	.8033622	-6.145417	.6916392	2.433651	1.060403	0.022	.8010166
**AT vs RT**	-1.969013	1.065104	.0855673	.9542685	-2.054581	1.447511	0.156	.962301
**AT vs CE**	1.233773	.8293501	.0451	.9695573	1.188673	1.267359	0.348	.9454061
**AT vs CT**	-1.993281	.3233369	-.6008282	.9795576	-1.392452	1.031071	0.177	.8886221
**RT vs CE**	2.380009	1.614384	1.223902	1.050782	1.156107	1.932302	0.550	.969296
**RT vs CT**	-1.42361	.7040041	1.562145	1.758234	-2.985755	1.881735	0.113	.9388633
**CE vs CT**	-2.189314	.8801987	-3.057759	.9253699	.8684449	1.259898	0.491	.9584703

HIIT, high-intensity interval training; AT, aerobic training; RT, resistance training; CE, combined aerobic and resistance exercise; CT, conventional therapy.

Supplementary Table 3. Local consistency test for 6MWD.

Side	Direct		Indirect		Difference			tau
	Coef.	Std. Err.	Coef.	Std. Err.	Coef.	Std. Err.	P>z	
**HIIT vs AT**	-37.9836	27.92976	-22.02895	34.09316	-15.95466	44.15886	0.718	31.93815
**HIIT vs CT**	-60.11888	31.54513	-75.71889	30.85149	15.60001	44.15902	0.724	31.93228
**AT vs RT**	5.112442	33.07192	7.372369	25.04828	-2.259928	41.62975	0.957	32.19323
**AT vs CE**	5.320438	31.25472	-9.190433	32.66799	14.51087	45.47395	0.750	32.15629
**AT vs CT**	-35.74707	13.83189	-40.06746	28.62339	4.320391	31.78129	0.892	32.03774
**RT vs CE**	15.89076	64.32548	-12.92993	28.97721	28.8207	70.64333	0.683	31.30936
**RT vs CT**	-37.70676	17.02982	-98.82847	57.58865	61.1217	60.2261	0.310	29.8238
**CE vs CT**	-27.20771	28.13513	-47.41712	35.9331	20.20941	45.97514	0.660	32.2303

HIIT, high-intensity interval training; AT, aerobic training; RT, resistance training; CE, combined aerobic and resistance exercise; CT, conventional therapy.

Supplementary Table 4. Local consistency test for SBP.

Side	Direct		Indirect		Difference			tau
	Coef.	Std. Err.	Coef.	Std. Err.	Coef.	Std. Err.	P>z	
**HIIT vs AT**	1.3	2.700643	-.5787118	4.220782	1.878712	5.010835	0.708	9.74e-10
**HIIT vs CT**	-.0799999	3.653877	1.798936	3.429409	-1.878936	5.011154	0.708	6.38e-09
**AT vs CE**	-3.500002	3.78929	-3.000252	6.486929	-.4997494	7.512581	0.947	3.27e-09
**AT vs CT**	.5734343	2.210195	-1.075143	3.849258	1.648577	4.438664	0.710	5.84e-09
**CE vs CT**	3.200008	6.175196	3.699592	4.278845	-.4995841	7.512752	0.947	1.59e-09

HIIT, high-intensity interval training; AT, aerobic training; RT, resistance training; CE, combined aerobic and resistance exercise; CT, conventional therapy.

Supplementary Table 5. Local consistency test for DBP.

Side	Direct		Indirect		Difference			tau
	Coef.	Std. Err.	Coef.	Std. Err.	Coef.	Std. Err.	P>z	
**HIIT vs AT**	.4000001	3.235007	-1.16554	3.611014	1.56554	4.848163	0.747	1.966446
**HIIT vs CT**	-.28	3.109004	1.286291	3.719966	-1.566291	4.848098	0.747	1.966431
**AT vs CE**	-3.7	2.225184	1.087782	3.060843	-4.787782	3.784198	0.206	1.12e-08
**AT vs CT**	1.514614	1.472755	-2.135442	2.493565	3.650056	2.896011	0.208	6.78e-07
**CE vs CT**	.1000161	2.742853	4.88765	2.607153	-4.787634	3.784236	0.206	7.58e-06

HIIT, high-intensity interval training; AT, aerobic training; RT, resistance training; CE, combined aerobic and resistance exercise; CT, conventional therapy.

Supplementary Table 6. Summary of adverse events.

Study	Type of intervention	Adverse events
Gjellesvik 2021	T: HIIT	1 death, 1 upper limb fracture after fall, 1 concussion, 1 hospitalization for femoral venous drainage
	C: CT	1 transient ischemic attack, 1aortic aneurysm, 1 minor hemorrhage, 1 seizure
Boyne 2016	T: HIIT	5 joint/muscle pain, 1 dizziness
	C: CT	1 joint/muscle pain
Hsu 2020	T: HIIT	1 hernia surgery, 1 recurrent stroke, 1 unstable blood pressure
	C: CT	1 recurrent stroke
Globas 2012	T: AT	1 hip pain, 2 falls
	C: CT	-
Mackay-Lyons 2013	T: AT	1 seizure
	C: CT	1 recurrent stroke, 1 brain tumor
Tang 2014	T: AT	11 falls
	C: CT	9 falls
Severinsen 2014	T1: AT	1 pain, 1 minor stroke
	T2: RT	-
	C: CT	-
Chang 2012	T: AT	1 aspiration pneumonia
	C: CT	1 recurrent cerebral infarction, 1epilepsy
Shao 2023	T: RT	1 deep vein thrombosis, 2 pneumonia, 4 fractures after falls
	C: CT	2 deep vein thrombosis, 4pneumonia

T, intervention group; C, control group; HIIT, high-intensity interval training; AT, aerobic training; RT, resistance training; CT, conventional therapy, -, not reported.
